# Boosting Coercivity of 3D Printed Hard Magnets through Nano‐Modification of the Powder Feedstock

**DOI:** 10.1002/advs.202407972

**Published:** 2024-10-22

**Authors:** Philipp Gabriel, Varatharaja Nallathambi, Jianing Liu, Franziska Staab, Timileyin David Oyedeji, Yangyiwei Yang, Nick Hantke, Esmaeil Adabifiroozjaei, Oscar Recalde‐Benitez, Leopoldo Molina‐Luna, Ziyuan Rao, Baptiste Gault, Jan T. Sehrt, Franziska Scheibel, Konstantin Skokov, Bai‐Xiang Xu, Karsten Durst, Oliver Gutfleisch, Stephan Barcikowski, Anna Rosa Ziefuss

**Affiliations:** ^1^ Technical Chemistry I and Center for Nanointegration Duisburg‐Essen (CENIDE) University of Duisburg‐Essen 45141 Essen Germany; ^2^ Max Planck Institute for Sustainable Materials 40237 Düsseldorf Germany; ^3^ Functional Materials, Institute of Material Science Technical University of Darmstadt 64287 Darmstadt Germany; ^4^ Physical Metallurgy, Institute of Material Science Technical University of Darmstadt 64287 Darmstadt Germany; ^5^ Mechanics of Functional Materials, Institute of Material Science Technical University of Darmstadt 64287 Darmstadt Germany; ^6^ Chair of Hybrid Additive Manufacturing, Ruhr‐University Bochum 44801 Bochum Germany; ^7^ Advanced Electron Microscopy Division, Institute of Material Science Technical University of Darmstadt 64287 Darmstadt Germany; ^8^ Department of Materials Imperial College London London SW7 2AZ UK

**Keywords:** additive manufacturing, grain refinement, laser ablation in liquids, nanoparticles, recapture powder melting, rich finite element simulations

## Abstract

The demand for strong, compact permanent magnets essential for the energy transition drives innovation in magnet manufacturing. Additive manufacturing, particularly Powder Bed Fusion of metals using a laser beam (PBF‐LB/M), offers potential for near‐net‐shaped Nd‐Fe‐B permanent magnets but often falls short compared to conventional methods. A less explored strategy to enhance these magnets is feedstock modification with nanoparticles. It is demonstrated that modifying a Nd‐Fe‐B‐based feedstock with 1 wt.% Ag nanoparticles boost the coercivity of the magnets to a record value of 935 ± 6 kA m^−1^ without further post‐processing or heat treatments. Suitable volumetric energy densities for the PBF‐LB/M process are determined using finite element simulations predicting melt pool behavior and part density. Microstructural analyses reveal finer grain sizes and more equiaxed nanocrystalline structures due to the modification. Atom probe tomography identifies three phases in the Ag‐modified samples, with Ag forming nanophase regions with rare‐earth elements near the amorphous Zr‐Ti‐B‐rich intergranular phase, potentially decoupling the Nd_2_Fe_14_B primary phase. The study shows that superior magnetic properties primarily result from microstructure modification rather than part density. These findings highlight inventive material design approaches via feedstock surface modification to achieve superior magnetic performance in additively manufactured Nd‐Fe‐B magnets.

## Graphical abstract

Innovative manufacturing using Laser Powder Bed Fusion (PBF‐LB/M) and 1 wt.% Ag nanoparticle modification boosts Nd‐Fe‐B magnets' coercivity to a record value of 935 ± 6 kA m^−1^, achieved without post‐processing or heat treatments. Microstructural analyses (SEM‐EBSD, HR‐TEM, atom probe tomography) reveal fine, equiaxed grains driving this enhancement, showcasing a very promising novel feedstock surface modification strategy.

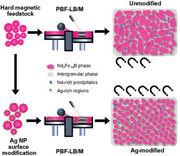

## 1 Introduction

Transitioning to electric vehicles and clean power generation requires strong and compact permanent magnets.^[^ ^1^, ^2^ ^]^ Currently, the strongest commercially relevant permanent magnets are made from alloys, including rare‐earth (RE) elements, particularly Nd‐Fe‐B magnets.^[^ ^2^ ^]^ However, the increasing demand for large quantities of RE poses challenges, generating delivery shortages and possibly volatile price fluctuations.^[^ ^1^, ^2^, ^3^ ^]^ Since the introduction of Nd‐Fe‐B magnets in 1984,^[^ ^4^ ^]^ scientists and corporations have been exploring methods to enhance their magnetic properties and optimize the usage of RE elements without compromising on mechanical and functional properties.^[^ ^3^, ^5^, ^6^ ^]^

One important option to achieve enhanced magnetic properties and possibly reduce material consumption is through the selection and optimization of the manufacturing technique. Recently, researchers started exploring the production of Nd‐Fe‐B magnets through additive manufacturing (AM).^[^ ^2^ ^]^ AM encompasses diverse emerging production techniques, which enable the creation of geometrically intricate and near‐net‐shaped parts, reducing the amount of material needed and allowing for shaping magnets according to their functional requirements.^[^ ^2^ ^]^ Producing fully dense and textured Nd‐Fe‐B magnets (similar to sintered magnets) without any non‐magnetic binder volumes, as they are required for polymer‐bonded magnets, is pursued to attain optimal magnetic properties.^[^ ^2^ ^]^

Powder bed fusion of metals using a laser beam (PBF‐LB/M) is a leading AM technique, extensively used for various alloys and applications, which allows for the small series production, offering high geometrical freedom, good dimensional accuracy, and reasonable surface finish.^[^ ^7^, ^8^, ^9^, ^10^ ^]^ PBF‐LB/M of different Nd‐Fe‐B‐based micro powders has already been reported in the literature, mainly focusing on laser processing parameters and their correlation to mechanical or magnetic properties.^[^ ^11^, ^12^, ^13^, ^14^, ^15^, ^16^, ^17^, ^18^, ^19^, ^20^, ^21^, ^22^, ^23^, ^24^ ^]^ Sufficient undercooling of the liquid melt is required for the direct nucleation of the Nd_2_Fe_14_B hard magnetic phase which otherwise can lead to the formation of larger volume fractions of the soft magnetic α‐Fe phase. Studies have reported that a lower volume fraction of the hard magnetic phase can result in significantly reduced magnetic properties.^[^ ^16^, ^25^ ^]^ However, recent findings of Tosoni et al.^[^ ^18^ ^]^ indicate that the high cooling rates in PBF‐LB/M are advantageous for Nd‐Fe‐B‐based permanent magnets, facilitating the formation of micro‐ and nanostructures in the as‐built parts, which are crucial for achieving superior permanent magnetic performance.^[^ ^5^ ^]^ Furthermore, Yao et al.^[^ ^24^ ^]^ demonstrated superior magnetic properties achieved after PBF‐LB/M processing compared to vacuum induction melting (VIM) and laser‐directed energy deposition (LDED) methods resulting from a higher volume fraction of the Nd_2_Fe_14_B phase and grain refinement observed in the microstructure (average grain sizes in the VIM‐processed, LDED‐processed and PBF‐LB/M‐processed samples were 6.7, 0.95, and 0.067 µm, respectively), both linked to the rapid cooling rates experienced (1.5 × 10^6^ K s^−1^) during solidification.

Another important route to improve permanent magnetic properties or reduce RE content is by minor alloy additions and compositional modifications. The ternary Nd_2_Fe_14_B (2‐14‐1) phase is pivotal for the overall good magnetic properties of Nd‐Fe‐B permanent magnets.^[^ ^26^ ^]^ However, the presence of a secondary phase, comprising a thin paramagnetic RE‐rich layer that surrounds and decouples the Nd_2_Fe_14_B grains, has been found to be highly relevant.^[^ ^3^, ^27^ ^]^ The chemical composition of this Nd‐rich boundary layer is similar to the Nd:Fe eutectic phase,^[^ ^28^ ^]^ but can be changed by alloying. Fuerst and Brewer^[^ ^29^ ^]^ analyzed the capabilities of multiple elements (Cd, Cu, Au, Ir, Mg, Ni, Pd, Pt, Ru, Ag, and Zn) to improve the permanent magnetic properties of Nd‐Fe‐B and found that especially the diamagnetic transition metals Ag, Cu, and Zn can enhance the coercivity significantly. Furthermore, the permanent magnetic properties of Nd‐based magnets have been shown to benefit from Ag additions during conventional sintering due to an increase in the volume fraction of the Nd‐rich phase.^[^ ^30^, ^31^ ^]^ The Ag addition to Nd‐Fe‐B‐based magnets also improved the fracture toughness,^[^ ^32^ ^]^ indicating its positive effect on mechanical properties. With Ag having a lower melting point than the Nd‐Fe‐B, it is melting rapidly and possibly filling open spaces in the loosely packed powder layers of the PBF‐LB/M process, potentially leading to a higher packing density, which positively affects the final part density^[^ ^33^ ^]^ and hence the magnetic performance. Minor additions of Ag enhancing the permanent magnetic properties, in general, is attributed to the compositional modifications observed with the Nd‐rich amorphous intergranular/grain boundary. The Ag enriched in the intergranular phase tends to improve the magnetic decoupling of the hard magnetic grains, thereby improving the coercivity. However, the understanding of the behavior of Ag nanoparticle addition to Nd‐lean MQP‐S feedstock material is lacking.

Following recent literature, introducing nanoparticles (NPs) into the feedstock of Al‐based powder serves as a means to provide nucleation sites for solidification, resulting in the formation of finer, more evenly shaped grains.^[^ ^34^, ^35^ ^]^ This method can be used as a tool for microstructure design of parts fabricated via PBF‐LB/M.^[^ ^7^, ^34^ ^]^ However, NP‐modified Nd‐Fe‐B powder feedstocks processed via PBF‐LB/M have not yet been reported. Importantly, the NPs used in PBF‐LB/M must be devoid of chemical precursors typically present after wet‐chemical synthesis, as they can negatively modify the composition of the NPs^[^ ^36^ ^]^ as well as impact the flow characteristics of powder feedstocks.^[^ ^37^, ^38^ ^]^ Additionally, organic residuals such as colloidal stabilizers bear the risk of vaporizing during processing, thereby causing unwanted balling effects, as it has been shown for laser direct‐writing of stabilizer‐containing Ag microparticle inks.^[^ ^39^ ^]^ Unlike wet chemical methods, laser ablation in liquids (LAL) requires neither chemical precursors nor organic surfactants.^[^ ^40^ ^]^ For LAL, a pulsed laser beam is focused on a solid metal target submerged in liquids, ablating material from the target surface with the removed material being collected in the surrounding liquid, and forming a fully inorganic colloidal dispersion of NPs.^[^ ^40^ ^]^

In this study, we modified the surface of Nd‐Fe‐B‐based micro ‐powder feedstock with 1 wt.% (equivalent to 0.7 vol.%) of laser‐generated Ag NPs to be used for PBF‐LB/M. By studying the impact of different process parameters on the melt pool formation during PBF‐LB/M processing experimentally and via finite element simulations for unmodified Nd‐Fe‐B feedstock, we defined the best parameter space. We then thoroughly investigated the impact of NP‐modification on as‐built part density and microstructure to reveal the impact of Ag NPs on the magnetic performance in comparison to unmodified samples.

## 2 Results and Discussion

Given the significant influence of processing parameters on the macroscopic properties of as‐built parts (e.g., part density and crack formation^[^ ^41^, ^42^, ^43^ ^]^), it is essential to establish ideal processing conditions before assessing the impact of NP‐based feedstock modification on part properties.

### 2.1 Impact of PBF‐LB/M Process Parameters on As‐Built Part Density and Melt Pool Formation

**Figure 1:**
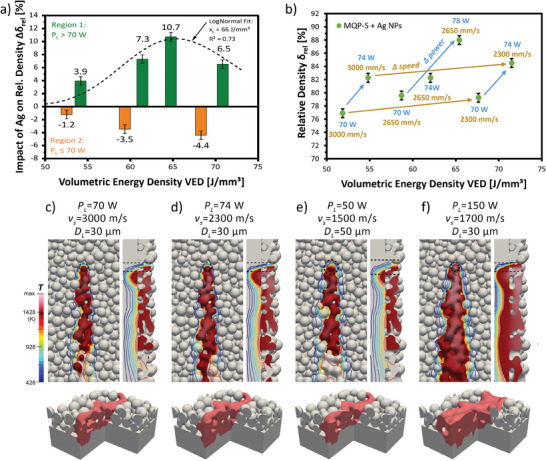
Impact of the applied VED (adjusted by changing **P**_**L**_ and **v**_**s**_) on the relative density **δ**_**rel**_ of as‐built magnets and the melt pool formation during processing. a) Increase of **δ**_**rel**_ of as‐built Ag‐modified samples compared to unmodified samples, if produced at **P**_**L**_ > 70 W (the LogNormal fit indicates an optimal VED for part density between 60–70 J mm^−^^3^). b) Impact of different **P**_**L**_ and **v**_**s**_ on the **δ**_**rel**_ of as‐built Ag‐modified samples. c–f) FE‐Simulations showing the impact of varying process parameters on the melt pool formation, heat distribution, and impacted powder particles. Overheated regions, where **T** >  **T**_**m**_, are drawn with a continuous color map, while areas with **T**  ≤  **T**_**m**_ are shown as isotherms. The laser spot characterized by **D**_**L**_ is indicated in dotted lines. All error bars are based on the accuracy of the analysis.

Thus, we started with a process parameter screening of PBF‐LB/M and determined an applicable parameter window of laser power *P_L_* ranging from 70 to 78 W, and scan speed *v_s_* from 2300 to 3000 mm s^−1^ (noted in the form of *P_L_*, *v_s_* hereafter), with a constant layer thickness of 30 µm and hatch distance of 15 µm and processed both, unmodified and Ag‐modified powder samples, within this parameter window (further details can be found in the materials and methods section and sections S1 and S2 of the Supporting Information). The resulting relative as‐built part densities (δ_*rel*_) for the unmodified parts varied between 75.0 and 83.7% and could be increased by up to 10.7 ± 0.7% through NP‐modification of the feedstock. Following **Figure** 1a and according to the literature^[^ ^9^ ^]^, the part density critically depends on the Volumetric Energy Density (VED), showing a clear maximum of δ_*rel*_ between 60 and 70 J mm^−^^3^. Note that the VED can be varied by a number of parameters, which underline interdependencies (details are given in the materials and methods section).^[^ ^11^, ^17^, ^44^, ^45^ ^]^

Our findings reveal that *δ*_*rel*_ can be improved by increasing *P_L_* or decreasing *v_s_*, as shown for the Ag‐modified as‐built parts in Figure 1b. Note that the unmodified as‐built parts follow the same trends. In both cases, the highest applied VED did not result in the highest *δ*_*rel*_, which follows a trend known from the literature.^[^ ^41^, ^46^ ^]^ While lower *v_s_* and higher *P_L_* are known to lead to higher *δ*_*rel*_ based on higher energy inputs leading to improved fusion of the powder particles, a too high *v_s_* and too low *P_L_* result in lower part densities because of insufficient energy input.^[^ ^11^, ^17^ ^]^ Nevertheless, too high energy inputs lead to defects and lower *δ*_*rel*_, e.g., delamination of consecutive layers due to insufficient heat transfer.^[^ ^8^ ^]^ But some studies reported that higher *P_L_* has a beneficial effect on the as‐built part density, e.g., Figure 20 in ref. [9]. Brown et al.^[^ ^47^ ^]^ investigated the correlation between *δ*_*rel*_ and *P_L_* on the Nickel alloy 625 and found that the average bulk density increased with increasing VED values, which is opposite to our findings in Figure 1b for as‐built parts produced at VED > 65 J mm^−^^3^. Overall, the *δ*_*rel*_ was increased by 10.7% via VED optimisation, yielding 88% relative density of the as‐built magnets.

### 2.2 FE‐Simulations of the PBF‐LB/M Process

To explain these apparent contradictions and extend the parameter window of the experiments, we investigated the effects of different process parameters on the melt pool behavior by recapturing the powder melting in finite element (FE) simulations of the PBF‐LB/M process, shown in Figure 1c–f. Detailed information on the modeling can be found in section 3 of the SI. The processing parameters, notably *P_L_*, *v_s_*, and the laser diameter, *D*_*L*_, are chosen as input parameters for the simulations of two selected combinations based on the experiments in this work (Figure 1c,d) and compared with two selected cases from literature as reference (Figure 1e ^[^ ^44^ ^]^ and Figure 1f ^[^ ^12^ ^]^).

In Figure 1c–f, the overheated region (*T* > *T*_*m*_), is noted with a continuous colormap. Cooler regions (*T*  ≤ *T*_*m*_) are indicated with temperature isotherms. Particles are fully molten within the overheated region, while partial melting or sintering must be considered in the cooler regions. The tendency of surface energy reduction drives mass transfer, notably surface diffusion and localized melt flow. Consequently, in regions with *T*  ≤ *T*_*m*_, the temperature is high enough to induce solid‐state sintering, as observed via the formation of necks between adjacent particles in Figure 1c–f. Comparing the different scenarios revealed that if *D*_L_ is held constant (Figure 1c,d,f: *D*_L_ = 30 µm), increasing *P_L_* or decreasing *v_s_* leads to greater heat accumulation at the beam spot, ultimately resulting in a more pronounced overheated region. A larger *D*_L_ focuses the laser energy on a larger area (2D) or volume (3D), which leads to a wider melt pool and more powder particles present in the heat‐affected zone, and hence possibly faster cooling. At fixed *v_s_*, high *P_L_* can lower part quality, mainly due to the reduction in part density via accompanied evaporation or even via keyholing that introduces extra pores by vaporization. Increased residual stress is also expected due to enhanced local temperature gradient around the melt pool. Therefore, the relationship between *P_L_* and δ_*rel*_ has a limited applicability for PBF‐LB/M of Nd‐Fe‐B‐based feedstocks, as factors, such as the *D*_L_, *v_s_*, powder bed properties, and further process parameters^[^ ^48^ ^]^ also have an influence.

The thermal‐microstructural simulations were performed assuming a complete power absorption within the laser spot, which has been shown to depend on *P_L_* and *D*_L_.^[^ ^49^, ^50^ ^]^ High‐fidelity ray‐tracing simulations also unveiled the positive linear correlation of the absorptivity on *P_L_* within a low VED range. When *P_L_* is high enough to create increased melt pool depth, more absorption events are expected due to the enhanced accommodation of reflections by the melt pool.^[^ ^50^ ^]^ Such an effect is, however, tentatively disregarded in the presented thermal‐microstructural simulations for variable control and should be explicitly implemented in future works.

Accordingly, the contradictions with the above‐described studies on 316L (1.4404) stainless steel^[^ ^9^ ^]^ or Nickel 625 (2.4856),^[^ ^47^ ^]^ can be explained by the influence of the Nd‐Fe‐B‐based material properties. The powder material's thermal properties are one factor that directly affects the observable process features and hence, the density and microstructure evolution of the material, together with the powder bed packing density, specific energy input, and laser diameter.^[^ ^49^, ^50^, ^51^ ^]^ For instance, melt pool size strongly relates to the powder materials' thermal conductivity.^[^ ^52^, ^53^ ^]^ The thermal conductivity of Nd‐Fe‐B is relatively low when compared to other typical alloys used in PBF‐LB/M processing,^[^ ^11^ ^]^ which leads to relatively low homogenized heat dissipation in the powder bed with the same powder size distribution.^[^ ^49^ ^]^

In summary, the simulations of Nd‐Fe‐B powder processing via PBF‐LB/M show a temperature field with a high‐gradient profile on the front and the bottom of the overheated region, implying a vast local heating/cooling rate and in‐process high thermal stress for all sets of processing parameters considered, as seen in Figure 1c–f. Processing Nd‐Fe‐B‐based feedstock with too high *P_L_* not only negatively affects part quality by reducing its density, as mentioned before, but also leads to an elongated microstructure, as shown in different studies.^[^ ^12^, ^44^ ^]^ Note that micro‐cracks and lack of fusion pores might cause the absence of anisotropy if they reduce the heat transfer in the building direction and influence the thermal gradient.^[^ ^21^ ^]^ However, we could successfully manufacture samples at appropriate VED, potentially with sintering contributing. The optimal range of VED to achieve high as‐built density has been found to be between 60 and 70 J mm^−^^3^.

### 2.3 Analysis of the Magnetic Properties of the As‐Built Parts

In the next step, we investigated the magnetic properties of the as‐built parts to find further correlations with the process parameters and the impact of the NP modification. For this field‐dependent magnetization curves were measured.

**Figure 2:**
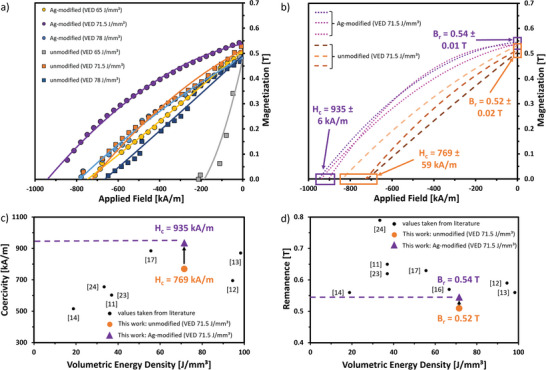
Analysis of magnetic properties of unmodified and Ag‐modified as‐built parts. a) Second quadrant of magnetic hysteresis loops of unmodified and Ag‐modified as‐built parts for different VED and b) direct comparison of three individual samples of unmodified (orange curves) and Ag‐modified (purple curves) as‐built parts after PBF‐LB/M using a VED of 71.5 J mm^−^^3^. c,d) VED and achieved magnetic properties of references (labeling as applied in this study and described in Section S5, Supporting Information) in comparison to our best results of unmodified (orange circle) and Ag‐modified (purple triangle) as‐built parts. Note that only results achieved with the MQP‐S feedstock, and without any kind of post‐processing have been included in the comparison.

The second quadrant of the resulting magnetic hysteresis loops is shown in **Figure** **2**a (the full hysteresis loops are shown in Figure S4, Section S4, Supporting Information) for as‐built parts (without further post‐processing) produced from unmodified and the Ag‐modified feedstock variant at different VEDs between 62 and 78 J mm^−3^. Figure 2b shows the hysteresis loops of the as‐built parts processed at the same VED of 71.5 J mm^−^^3^ (74 W and 2300 mm s^−1^), resulting in the highest magnetic properties for the Ag‐modified feedstock (purple lines) with a coercivity *H*_*c*_ of 935 ± 6 kA m^−1^ and a remanence *B*_*r*_ of 0.54 ± 0.01 T. For the unmodified feedstock (orange lines), a *H*_*c*_ of 769 ± 59 kA m^−1^ and a *B*_*r*_ of 0.52 ± 0.02 T were measured. We found that the Ag‐modification of MQP‐S significantly increases the coercivity by roughly 17 ± 6% without negatively affecting the remanence. Also, the standard deviation of both magnetic properties is reduced, and the squareness of the hysteresis loops is slightly improved. Note that we expected the inclusion of Ag to reduce the remanence as it is a diamagnetic material, but the amount of required NPs is small enough to have a fairly low impact on the remanence. Also, the results at a VED of 65 J mm^−^^3^, shown in Figure 2a, are worth noting, as the unmodified sample (silver line) only reached a coercivity *H*_*c*_ of roughly 175 kA m^−1^, whereas the corresponding Ag‐modified sample (yellow line) led to roughly 750 kA m^−1^. As the unmodified sample only shows a few measurement points in the second quadrant, the results are too indecisive to draw conclusions, and this must be investigated further.

Comparing our results with literature data on PBF‐LB/M of MQP‐S, the highest achieved coercivity *H*_c_ for the Ag‐modified as‐built part amounts to 935 ± 6 kA m^−1^. This is, to our best knowledge, the highest coercivity achieved by PBF‐LB/M processed MQP‐S‐based feedstocks without post‐processing, e.g. heat treatments, exceeding literature values as shown in Figure 2c. Note that significant alterations of MQP‐S or different compositions of Nd‐Fe‐B‐based feedstock have not been included in the comparison as they lack comparability. Plotting the VEDs versus the achieved magnetic properties of references (labeling as applied in this study and described in Section S5, Supporting Information) our results show possible trends. Still, as the data pool is insufficient, we refrain from drawing a guide to the eyes. Here, the lower orange point indicates the achieved properties with unmodified MQP‐S, and the upper purple point indicates the achieved results by adding 1 wt.% Ag NPs, where both have been produced at the same VED of 71.5 J mm^−^^3^. Multiple studies reached higher remanence results, as shown in Figure 2d, with Yao et al.^[^ ^24^ ^]^ reaching the highest value of 0.79 T, which could be connected to their investigation of a different hatch distance of 100 µm potentially influencing the solidification rate of neighboring melt pools leading to slightly better grain alignment and texture, but also the α‐Fe content, which on the other hand led to the coercivity of 656 kA m^−1^ as shown in Figure 2c.

Furthermore, comparable values of VED can be achieved by completely different process parameter combinations, which might affect the solidification and microstructure. Accordingly, it is important to better understand how the process parameters and the addition of Ag NPs lead to these magnetic properties and how they affect the microstructure and chemical composition, which has been investigated in the next steps.

### 2.4 Microstructural Analysis: Morphology, Size, Orientation, and Composition of Grains

**Figure 3:**
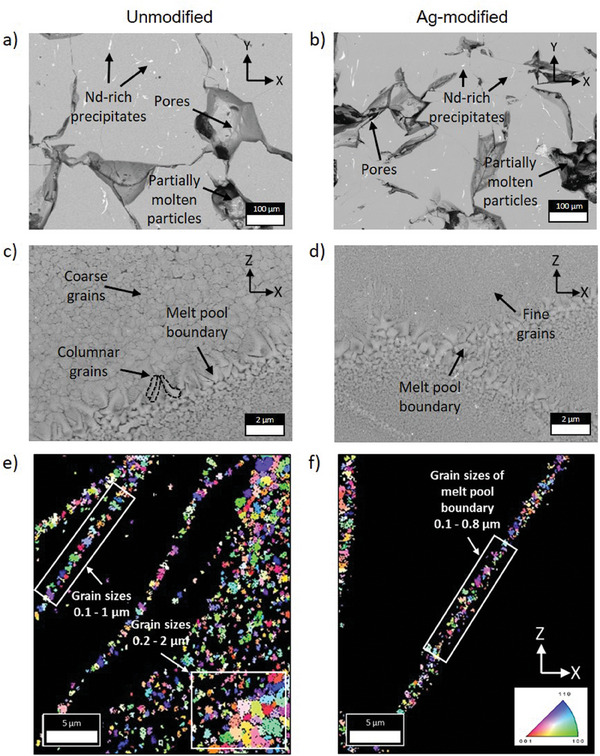
Microstructural analysis of unmodified and Ag‐modified as‐built parts after PBF‐LB/M at a VED of 71.5 J mm^−^^3^. a,c) SEM images of the as‐built unmodified and b,d) of the Ag‐modified samples taken across horizontal (X‐Y) and vertical (X‐Z) cross‐sections to the building direction, respectively. e,f) Inverse pole figure (IPF) maps analyzed by EBSD of unmodified and Ag‐modified samples, respectively, with a VED of 71.5 J mm^−^^3^. Cross‐sections along the X‐Z plane have been made, with the Z‐axis resembling the build direction. The crystallographic orientations are indicated by the orientation legend for tetragonal symmetry.

The structural characterization from the micro down to the atom scale employed SEM‐EBSD, TEM‐EDS, and APT (details are given in the materials and methods section). The microstructure of the unmodified and Ag‐modified as‐built parts, which have been produced at a VED of 71.5 J mm^−^^3^ (74 W and 2300 mm s^−1^), was examined via SEM, **Figure** **3**a,b. Arbitrarily shaped micropores and partially molten particles confirm the initially discussed degree of density in the as‐built samples, possibly originating from the low thermal conductivity of the feedstock material combined with the high cooling rates. Besides, the BSE‐SEM images show characteristic melt pool boundaries with larger columnar grains gradually shifting toward polygonal grains inside the melt pool area.^[^ ^54^ ^]^ The core of the melt pool consists of more equiaxed/polygonal grains that become irregular closer to the melt pool boundary, as can be seen in Figure 3c,d. This layered microstructure of the melt pools repeats itself across the build direction (Z‐axis).

The unmodified and Ag‐modified samples show significant differences in the grain structure of the melt pool region. The Ag‐modified sample shows a much finer equiaxed grain structure distribution than the unmodified sample with irregular polygonal grains, as highlighted in Figure 3c,d. This indicates that Ag NPs play a role in the grain refinement of the melt pool microstructure, resulting in finer grain size. EBSD was performed for both microstructures to study the grain size distribution and orientation differences. Figure 3e,f show the inverse pole figure (IPF) maps analyzed by EBSD of the as‐built unmodified and Ag‐modified samples. Both show non‐indexed regions appearing black, which originate from grain sizes below the detection limit of EBSD. The microstructure of the unmodified sample shows regions of fine grains in the melt pool boundary in the size of roughly 0.1–1.0 µm and coarser grains in the range of 0.2–2 µm size (Figure 3e). In contrast, the microstructure of the Ag‐modified sample shows only fine grains in the range of 0.1–0.8 µm (Figure 3f), which are in the melt pool boundary regions. There seems to be no texture as the grains of both sample types are orientated arbitrarily in all possible axis directions.

SEM‐EDS was performed to gather composition information of the melt pool region (shown in Figures S6–S9, Supporting Information). Here, we identified a Nd‐Fe‐B phase with a composition close to the expected 2:14:1 stoichiometry, within the accuracy achievable of EDS. This phase appears in grey in the BSE‐SEM images, whereas Nd‐rich precipitates, appear with a bright contrast (see Supporting Information for details).

**Figure 4:**
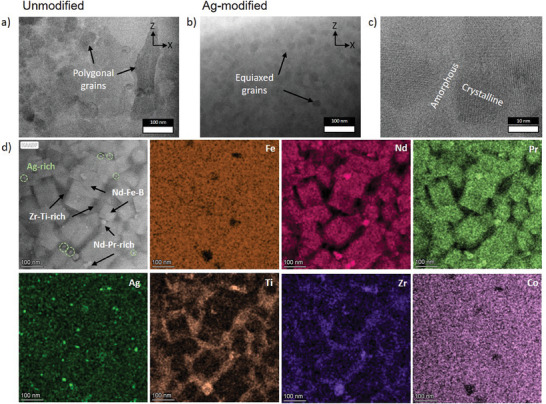
Microstructural and compositional analysis via HR‐TEM and STEM‐EDS. a) HR‐TEM bright field image of the unmodified sample; b,c) of the Ag‐modified sample; d) STEM‐EDS mappings of the Ag‐modified sample. Exemplary Ag‐rich regions are highlighted in dotted light green circles in the STEM‐HAADF image (d). All images were taken on the vertical cross‐sections (X‐Z plane) representing the PBF‐LB/M build direction.

Since the performance‐enhancing microstructural features are on a finer scale, HR‐TEM and STEM‐EDS were performed on vertical cross‐sections (X‐Z plane) of unmodified and Ag‐modified samples, **Figure** **4**. The high cooling rates during the PBF‐LB/M process result in a nanograined microstructure. The unmodified sample exhibits elongated and irregular polygonal grains with a high aspect ratio in the microstructure (Figure 4a), similar to the features observed in the BSE‐SEM micrographs (Figure 3c,d). The grain sizes vary largely, ranging from 50 nm to a few hundred nanometers with an average diameter of 65 ± 21 nm (diameter equivalent). On the other hand, the Ag‐modified sample (Figure 4b) shows a finer grain size distribution with an average grain size of 28 ± 7 nm at the melt pool region. The grains also seem to be equiaxed, indicating the occurrence of grain refinement during solidification.^[^ ^55^, ^56^ ^]^ A magnified view of the equiaxed grain distribution of the Ag‐modified sample is shown in Figure 4c. The primary crystalline Nd_2_Fe_14_B phase can be seen surrounded by a thin amorphous intergranular phase (for further XRD and STEM‐HAADF data, proving the 2:14:1 crystalline phase, see Figures S12–S14, Supporting Information). Figure 4d shows the individual STEM‐EDS elemental maps recorded close to the melt pool boundary of the Ag‐modified sample. The primary phase regions are the Nd‐rich, Nd_2_Fe_14_B phase, surrounded by a Ti‐ and Zr‐rich intergranular phase, with Fe being distributed in both phases. In addition, Nd‐Pr‐rich precipitate regions and Ag‐rich regions can be found distributed across the microstructure. Overall, the HR‐TEM micrographs of both samples provide evidence of a nanocrystalline microstructure after PBF‐LB/M as an added advantage compared to conventional processing methods.

**Figure 5:**
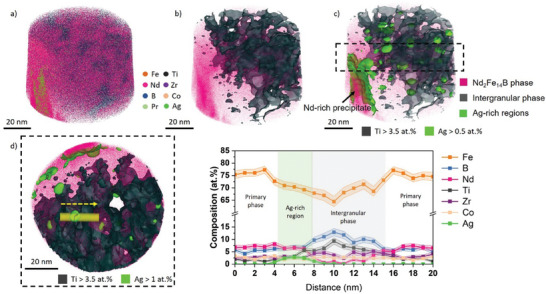
APT results of the Ag‐modified, additively manufactured (PBF‐LB/M) magnet sample. a) Section of the 3D reconstructed APT data; b) 3D atom map showing the intergranular phase with the isosurface of Ti (grey) > 3.5 at.%; c) 3D atom map showing the Ag distribution with the isosurface of Ag (light green) > 0.5 at.% in addition to the intergranular phase; d) 1D composition distribution of elements across the primary phase regions with a Ag‐rich and the intergranular phase in between.

Finally, we used APT to obtain the nanoscale distribution of Ag and other elements in the different phases of the Ag‐modified sample. **Figure** **5**a shows a section of the 3D reconstructed APT dataset of a specimen prepared from the center of the melt pool region of the Ag‐modified sample (the whole reconstruction is shown in Figure S14, Supporting Information). Ti‐, B‐, and Zr‐rich regions can be seen separating the Nd_2_Fe_14_B phase. The Ti‐, B‐, and Zr‐rich regions form the intergranular phase observed in the microstructure. The intergranular phase is Nd‐depleted, characteristically observed for the Nd‐lean MQP‐S feedstock after PBF‐LB/M processing.^[^ ^23^ ^]^ An Nd‐, Pr‐, and Ag‐rich precipitate region can also be identified on the bottom left (Figure 5).

We used isosurfaces with Ti concentration greater than 3.5 at.% (based on the Ti composition distribution in the intergranular phase) to illustrate the distribution of the primary and the intergranular phase regions in the reconstructed data. These isosurfaces represent the distribution of the interconnected intergranular phase regions that effectively separate the magnetic primary Nd_2_Fe_14_B phase. An isosurface with Ag concentration greater than 0.5 at.% is superimposed onto the point cloud as well in Figure 5c, which highlights, in light green, the distribution of Ag in the melt pool core of the sample. Besides the enrichment in the Nd‐rich precipitate, Ag is agglomerated into nanoscale‐sized regions distributed in the intergranular phase, often abutting the primary Nd_2_Fe_14_B phase. It indicates that the Ag‐rich regions play a direct role in the microstructure's restructuring during the melt pool's solidification. The 1D composition profile across different phases along a 5‐nm‐diameter cylindrical region of interest (ROI) is plotted in Figure 5d. The cylindrical ROI passes from one primary Nd_2_Fe_14_B phase to the next through an Ag‐rich region and the intergranular phase layer. The Nd_2_Fe_14_B phase primarily consists of Fe, Nd, and B in addition to low amounts of Pr, Co, Zr, and Ti. In contrast, the intergranular phase is enriched in Ti, B, and Zr with relatively low amounts of Fe and nearly no Nd compared to the primary phase. Co seems to be equally distributed across both phases. No noticeable amount of Ag concentration is found in the primary or intergranular phases. Trace amounts of Cu, Si, and Cr were also found in the reconstructed dataset. The measured values are given in Table S6, Supporting Information.

### 2.5 Physicochemical Mechanism Behind the Ag Nanoparticle Modification

**Figure 6:**
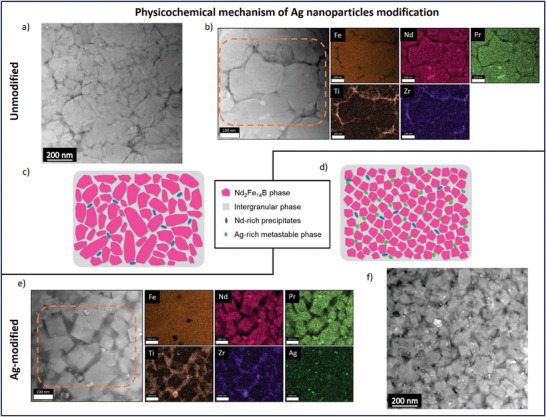
Comparison and schematic overview of the underlying physicochemical mechanism of Ag nanoparticles affecting the formation of microstructure, elemental composition, and phases in as‐built Nd‐Fe‐B‐based samples produced via PBF‐LB/M at a VED of 71.5 J mm^−3^. (a–c) show the representative STEM‐HAADF, STEM‐EDS mapping, and schematic representation of the phase composition in the microstructure of the unmodified sample respectively. (d–f) show the schematic representation, STEM‐EDS mapping and STEM‐HAADF image highlighting the phase composition in the microstructure of the Ag‐modified sample respectively.

A detailed comparison and schematic overview of the underlying physicochemical mechanism of Ag modification on the microstructure formation, elemental, and phase composition in as‐built Nd‐Fe‐B‐based samples produced via PBF‐LB/M is shown in **Figure** **6**. The unmodified samples exhibit elongated and irregular polygonal grains with a high aspect ratio in the microstructure (shown in the HR‐TEM/STEM images in Figures 4a and 6a; Figure S13, Supporting Information). On the other hand, the Ag‐modified samples show equiaxed fine‐grained microstructure indicating the occurrence of grain refinement during solidification (shown in the HR‐TEM/STEM images in Figures 4b and 6f; Figure S14, Supporting Information). The rise in coercivity with smaller grain sizes is linked to the reduction of the stray field generated by neighboring grains.^[^ ^57^ ^]^ For the nanocrystalline grains which are smaller than the size of single domains (≈250 nm) Bloch walls cannot develop and the impact of the domain walls^[^ ^6^ ^]^ and the exchange‐coupled effect, especially in nanocomposite hard magnets^[^ ^58^ ^]^, becomes increasingly important for the magnetic properties. Fischer et al.^[^ ^58^ ^]^ determined numerically that a nanocrystalline exchange‐coupled microstructure with more equiaxed regular‐shaped grains improves the magnetic properties due to improved distribution of the soft and hard magnetic phases.

Our in‐depth microstructural and compositional analysis revealed the phase mixture of the rapidly solidified as‐built samples to be the primary magnetic phase, Nd_2_Fe_14_B surrounded by a Ti‐Zr‐rich intergranular phase in addition to the random distribution of Nd‐Pr‐rich precipitates. However, for the Ag‐modified sample, additional nanosized phase regions are observed where the Ag is found to be enriched along with the presence of other elements like Fe, Nd, B, etc. STEM‐HAADF and STEM‐EDS (Figures 4 and 6; Figure S14, Supporting Information) analyses indicate that Ag is present mostly in the nanosized phase with no noticeable dissolution of Ag in the primary or the intergranular phase indicated by APT analysis. Also to be noted is the distribution of the Ag‐rich phase being adjacent to the primary Nd_2_Fe_14_B phase regions. Nucleation of metastable phases under rapid cooling conditions of the liquid melt in the Nd‐Fe‐B system has been reported earlier.^[^ ^59^, ^60^ ^]^ The nanosized Ag‐rich phase regions observed mimic the occurrence of metastable phase nucleation under the rapid solidification conditions during the PBF‐LB/M process. The elemental compositions observed for the Ag‐rich phase from APT match closely to the Nd_2_Fe_17_B_x_ (x  =  0–1) metastable phase which is reported to solidify directly from the liquid melt preceding the nucleation of the primary Nd_2_Fe_14_B phase under fast cooling conditions.^[^ ^59^ ^]^ The microstructural observations of the Ag‐modified as‐built parts indicate the initial nucleation of the Ag‐rich metastable phase that acted as additional nucleation sites aiding in the heterogeneous nucleation of the primary Nd_2_Fe_14_B grains resulting in a finer, more equiaxed microstructure thereby improving the permanent magnetic properties. Furthermore, the reheating effects from printing consecutive melt tracks next to and on top of each other during the PBF‐LB/M process could potentially alter the composition, structure, and distribution of the metastable phases after solidification. However, while further computational thermodynamic and non‐equilibrium solidification calculations are required to fully rationalize the formation of the metastable Ag‐rich phase, it appears to be a valid hypothesis for the current system. Focusing on systematic investigation of the nanoparticle‐modified powder's magnetic properties and related microstructural evolution during PBF‐LB/M processing, these additional calculations and measurements are beyond the scope of our current study.

## 3 Conclusion

This study presents advancements in the optimization of process parameters for hard magnets produced via PBF‐LB/M, supported by finite‐element simulations. A significant innovation is the surface modification of the Nd‐Fe‐B‐based micro powder feedstock by the addition of 1 wt.% Ag nanoparticles, positively affecting the microstructure formed during the PBF‐LB/M process. The Ag‐modified sample exhibits a 17 ± 6% increase in coercivity compared to the unmodified counterpart, without sacrificing remanence, even without further post‐processing. The addition of Ag refines the microstructure through the formation of a metastable Nd_2_Fe_17_B_x_ (x = 0–1) phase that acts as heterogeneous nucleation sites for the formation of finer, more equiaxed Nd_2_Fe_14_B primary grains resulting in the coercivity enhancement. Our findings highlight the critical role of the microstructure in controlling the magnetic properties, and surface modifications with minute amounts of Ag nanoparticles bear promises for further enhancements of the properties of additively manufactured hard magnets.

## 4 Experimental Section

### Characterization of the Nd‐Fe‐B‐Based Micro Powder Feedstock MQP‐S

This study used a gas‐atomized Nd‐Fe‐B‐based isotropic powder manufactured by Magnequench (Tuebingen, Germany). The powder is commercially known as MQP‐S‐11‐9‐20001 (referred to as MQP‐S throughout this study) with a nominal chemical composition of Nd_7.5_Pr_0.7_Zr_2.6_Ti_2.5_Co_2.5_Fe_75.4_B_8.8_,^[^ ^61^ ^]^ which was sieved to a particle size distribution (PSD) of 20–64 µm, better resembling the typical PSD for the PBF‐LB/M process, with the arithmetic mean *µ*_*d*_  =  32 µm and standard deviation *δ*_*d*_  =  8.7 µm. A surface‐weighted size distribution can be found in Figure S1a (Supporting Information). It has a spherical morphology and is generally used for manufacturing bonded magnets, particularly by injection molding, extrusion, and calendaring. In powder condition, it achieved a coercivity *H*_*c*_ of up to 730 kA m^−1^ and a remanence *B*_*r*_ of up to 0.75 T, according to the supplier's material data sheet.^[^ ^61^ ^]^ Characterization of the powder size distribution and morphology was performed by scanning electron microscopy (SEM, Philips ESEM‐XL30 FEG, 20 kV). The micro powder density was measured to be 7.4 g cm^−^^3^ using a pycnometer (Borosilicate glass 3.3. DIN ISO 3507, BRAND 25 ml type Gay‐Lussac).

### Laser‐Based Synthesis of Ag NPs and Surface Modification of the MQP‐S Feedstock

The Ag NPs were prepared by pulsed laser ablation in liquids (LAL),^[^ ^40^ ^]^ where the laser beam ablated the surface of a bulk Ag target (20 mm (width) x 80 mm (length) x 2 mm (thickness)) submerged in deionized water, using an ns‐pulsed laser (Innoslab, IS400‐1‐L, Edgewave GmbH, Würselen, Germany) at 145 W average output power. The laser wavelength was 1064 nm, with a laser pulse duration of 8 ns and a pulse repetition rate of 5 kHz (resulting in a pulse energy of 29 mJ). A galvanometer scanner (intelliSCAN‐20, Scanlab AG, Purchheim, Germany) with a scan speed of 2 m s^−1^ and an F‐theta lens focusing optics f = 100 mm was employed to move the laser beam over the target surface. A flow‐through ablation chamber was used (as described in ref. [62]). The ablation was done in deionized water with the addition of 0.1 µg L^−1^ NaOH, which is known to be a good inorganic stabilizer.^[^ ^63^ ^]^ The hydrodynamic diameter of the synthesized Ag NPs was measured by analytical disc centrifugation (ADC, CPS instruments) at a centrifugation speed of 24 000 rpm with a lower detection limit of 5 nm (Figure S1b, Supporting Information).

The modification of MQP‐S micro powders’ surface with 1 wt.% (corresponds to roughly 0.7 vol.%) of Ag NPs was performed by mixing the MQP‐S powder directly with the colloid of Ag NPs in water, which corresponds to a theoretical covered surface of 50%, or half a monolayer (see Figure S1c, Supporting Information). To force electrostatically‐controlled support,^[^ ^64^ ^]^ the pH value of the Ag NPs was lowered to 7, which is a value between the isoelectric point of MQP‐S and Ag NPs, but still high enough to avoid a negative effect on the metal powder surface. After the NPs were successfully deposited on the surface of the MQP‐S, the modified feedstock was separated from the liquid by centrifugation (4000 rpm at 10°C for 15 minutes) and dried in a vacuum oven at 40°C for 12 h.

### As‐Built Part Production via Laser Powder Bed Fusion (PBF‐LB/M)

A TruPrint 1000 by Trumpf (laser beam wavelength at 1070 nm and a focal diameter of 30 µm) was used as the PBF‐LB/M system for as‐built part production under Ar atmosphere. Cylindrical parts with a diameter and height of 5 mm were fabricated directly on a build platform composed of C45 steel (see Figures S2 and S3, Supporting Information). 15 samples have been used for further evaluation. The applied volumetric energy density (VED) is calculated as follows:

(1) $$VED=\frac{P_{L}}{v_{s}\cdot h\cdot LT}$$

with *P*_L_ being the laser power, *v*_s_ being the scan speed, *h* being the hatch distance and *LT* being the layer thickness (throughout this manuscript referred to as VED, *P*_L_, *v*_s_, *h*, and *LT*, respectively). It is a synthetic parameter typically used in [J mm^−^^3^], which cannot be considered an absolute indicator due to its limit in capturing the complexity of the melt pool physics of PBF‐LB/M, as sufficiently discussed in the literature.^[^ ^65^ ^]^ However, VED found its utility in explaining the effects of process parameters on part properties like surface roughness, hardness, and porosity characteristics.^[^ ^66^ ^]^ As it is a value often used in the context of PBF‐LB/M, it is applied and finite element‐simulated in this study to enable a comparison of our results with published data of other groups who manufactured magnetic as‐built parts via PBF‐LB/M. Section S3 (Supporting Information) gives a detailed description of the applied model and parameters of the FE simulation of the PBF‐LB/M process to recapture melting. Additionally, the VED is employed in this study to classify the impact of adding Ag NPs produced with the same parameter combinations. Because MQP‐S is not optimized for high melting temperatures (tends to lose hard magnetic phase), PBF‐LB/M was performed at moderate process conditions of VED. These have been adjusted via the *P*_L_ in increments of 4 W, ranging from 70 to 78 W, as well as varying the *v*_s_ in increments of 350 mm s^−1^, ranging from 2300 to 3000 mm s^−1^. All as‐built parts were manufactured with a constant *LT* of 30 µm and a constant *h* of 15 µm.

### Analysis of the As‐Built Parts’ Magnetic Properties

To determine the magnetic performance of the manufactured as‐built parts, isothermal magnetization measurements were performed in the as‐built state without further post‐processing steps using a physical property measurement system vibrating sample magnetometer (PPMS‐VSM, Quantum Design PPMS‐14) at 294 K under an applied magnetic field of up to ± 3 T to ensure magnetic saturation. Three different as‐built parts per feedstock (modified with Ag NPs and unmodified) and VED were analyzed with an accuracy of the measurements of ± 1%.

### Analysis of the As‐Built Parts’ Density, Phases, and Microstructure

In this study, the Archimedean density of all as‐built parts was measured following DIN EN ISO 2738 with an accuracy of the measurements of ± 0.67%, which led to the error bars given in Figure 1a. The chemical and structural characterization of the as‐built parts produced at a VED of 71.5 J mm^−3^ was carried out via scanning electron microscopy (SEM, TESCAN VEGA3) by backscattered electron (BSE) imaging and energy‐dispersive X‐ray spectroscopy (EDS) point scans and mappings using 20 kV acceleration voltage. To obtain the grain orientation, electron backscatter diffraction (EBSD) analyses of at least three samples each were also performed using the field emission gun scanning electron microscopy (FEG‐SEM, TESCAN MIRA3), operating at 30 kV with a step size of 40 nm. For each sample, two maps with an area of 24×54 µm^2^ have been analyzed. To improve the indexing rate, a neighbor pattern averaging, and re‐indexing (NPAR) post‐processing routine was applied using the software OIM Analysis 8.6 (EDAX). Grains are induced only from a minimum number of 10 pixels. Qualitative phase analysis was performed using X‐ray diffraction (XRD) measurements for unmodified and Ag‐modified as‐built samples and unmodified MQP‐S powder (Rikaku Smartlab 9 kW diffractometer) with Cu Kα radiation (λ = 0.15406 nm), where the angle 2θ was varied between 20° and 90° with a step size of 0.01° and a scan speed of 1°/min, and the voltage and current used for the experiment were 45 kV and 200 mA, respectively. Rietveld refinement of the XRD patterns was performed using the BRUKER TOPAS Version 5.0 software to calculate the lattice parameters of the tetragonal Nd_2_Fe_14_B phase. More in‐depth nanostructural analysis has been carried out on three samples each of the Ag‐modified and unmodified samples, using scanning electron microscopy – energy dispersive X‐ray spectroscopy (SEM‐EDS) maps and point scans as well as high‐resolution transmission and scanning transmission electron microscopy imaging (HR‐TEM and STEM, JEM ARM 200F, by JEOL, combined with the EDS‐detector JED 2300T, by JEOL). The lattice structure and distribution of elements in different phases were studied using scanning transmission electron microscopy – high‐angle annular dark field imaging combined with STEM‐EDS (Thermo Fischer Titan Themis 300 – probe corrected). TEM specimens and needle‐shaped specimens for atom probe tomography (APT) were prepared using a dual beam scanning electron microscope – Ga‐ion focused ion beam (SEM‐FIB) microscope (Thermo Fischer Helios 600i). APT measurements of Ag‐modified samples were carried out using a local electrode atom probe (LEAP 5000 XR, by Cameca Instruments) with a laser pulse energy of 50 pJ, a laser frequency of 125 kHz, a detection rate of 0.5% at a base temperature of 40 K. Data reconstruction and analysis was done using the software AP Suite by Cameca Instruments following the voltage‐based reconstruction protocol.

## Conflict of Interest

The authors declare no conflict of interest.

## Author Contributions

P.G. and V.N. contributed equally to this work. P.G. designed the methodology; performed validation; visualized the idea for the study; contributed to original draft preparation; and wrote, reviewed, and edited the manuscript. V.N. performed validation; visualized the idea for the study; contributed to original draft preparation; and wrote, reviewed, and edited the manuscript. J.L., F.S., T.D.O., Y.Y., N.H., E.A., O.R.‐B, and Z.R. performed validation and wrote, reviewed, and edited the manuscript. L.M.‐L., J.S., and B.‐X.X. performed project administration and wrote, reviewed, and edited the manuscript. B.G. performed project administration and validation; visualized the idea for the study; wrote, reviewed, and edited the manuscript. K.S., F.S., and K.D. performed project administration and validation, and wrote, reviewed, and edited the manuscript. O.G. performed funding acquisition, project administration, and supervision, and wrote, reviewed, and edited the manuscript. S.B. conceptualized the idea for the study; performed funding acquisition; designed methodology; performed project administration, supervision, and validation, and wrote, reviewed, and edited the manuscript. A.R.Z. conceptualized the idea for the study; designed the methodology; performed project administration, supervision, and validation; visualized the idea for the study; and wrote, reviewed, and edited the manuscript.

## Supporting information



Supporting Information

## Data Availability

The data that support the findings of this study are available from the corresponding author upon reasonable request.
