# Supplementary material for: Boosting Coercivity of 3D Printed Hard Magnets through Nano‐Modification of the Powder Feedstock
Source: Adv Sci (Weinh). 2024 Oct 22;11(46):2407972. doi: 10.1002/advs.202407972 (PMC11633474; doi:10.1002/advs.202407972)
Supplement: Supplementary file 1 — Supporting Information [file ADVS-11-2407972-s001.docx]

Supporting Information

**Boosting coercivity of 3D printed hard magnets through nano-modification of the powder feedstock**

*Philipp Gabriel^#^, Varatharaja Nallathambi^#^, Jianing Liu, Franziska Staab, Timileyin David Oyedeji, Yangyiwei Yang, Nick Hantke, Esmaeil Adabifiroozjaei, Oscar Recalde-Benitez, Leopoldo Molina-Luna, Ziyuan Rao, Baptiste Gault, Jan T. Sehrt, Franziska Scheibel, Konstantin Skokov, Bai-Xiang Xu, Karsten Durst, Oliver Gutfleisch, Stephan Barcikowski*, Anna Rosa Ziefuss*

P. Gabriel, V. Nallathambi, S. Barcikowski, A. R. Ziefuss

Technical Chemistry I and Center for Nanointegration Duisburg-Essen (CENIDE)

University of Duisburg-Essen

45141 Essen, Germany

E-mail: stephan.barcikowski@uni-due.de

V. Nallathambi, Z. Rao, B. Gault

Max Planck Institute for Sustainable Materials

40237 Düsseldorf, Germany

J. Liu, F. Scheibel, K. Skokov, O. Gutfleisch

Functional Materials, Institute of Material Science

Technical University of Darmstadt

64287 Darmstadt, Germany

F. Staab, K. Durst

Physical Metallurgy, Institute of Material Science

Technical University of Darmstadt

64287 Darmstadt, Germany

T. D. Oyedeji, Y. Yang, B.-X. Xu

Mechanics of Functional Materials, Institute of Material Science

Technical University of Darmstadt

64287 Darmstadt, Germany

N. Hantke, J. T. Sehrt

Chair of Hybrid Additive Manufacturing

Ruhr-University Bochum

44801 Bochum, Germany

E. Adabifiroozjaei, O. Recalde-Benitez, L. Molina-Luna

Advanced Electron Microscopy Division, Institute of Material Science

Technical University of Darmstadt

64287 Darmstadt, Germany

B. Gault

Department of Materials

Imperial College London

London, UK

^#^ equal contribution

**1. Production of Ag-modified feedstock material for PBF-LB/M**

**1.1 Laser ablation in liquids process**

The technique of laser ablation in liquids (LAL) was first described by A. Fojtík and A. Henglein to synthesize surfactant-free nanoparticles (NPs) easily and rapidly.^[1]^ In contrast to wet chemical methods, laser synthesis does not demand the employment of chemical precursors and organic surfactants. LAL requires the delivery of a high energy density to the target material to overcome the ablation threshold and promote material removal from the target surface. The removed material is collected in the surrounding liquid forming a colloidal dispersion of NPs. To overcome the ablation threshold while reducing the laser's interaction with the surrounding liquid, short and ultrashort laser pulses in the range of fs up to ns are employed instead of continuous wave lasers that suffer from strong heating of the liquid. The LAL process outcome is determined by a variety of parameters, including material and laser features. Another advantage of LAL is the reduction of byproducts in the colloids, allowing the direct employment of the generated NPs without further purification steps. This is particularly appropriate for the Ag NPs generated in the current work.

**1.2 Additivation of Ag NPs on MQP-S microparticles**

The additivation of Ag NPs on the MQP-S microparticles (see details of MQP-S in the materials and methods section of the main manuscript) surface was performed by directly mixing the MQP-S powder with the Ag NP colloid. To force diffusion-controlled supporting, the pH value of the colloid was lowered to 7, which is a value between the isoelectric points of MQP-S and Ag NPs, but still high enough to avoid any negative effect on the metal powder surface. After the NPs were successfully deposited on the surface of the MQP-S, the modified feedstock was separated from the liquid by centrifugation (4,000 rpm at 10˚C for 15 minutes) and dried in the vacuum oven at 40˚C.


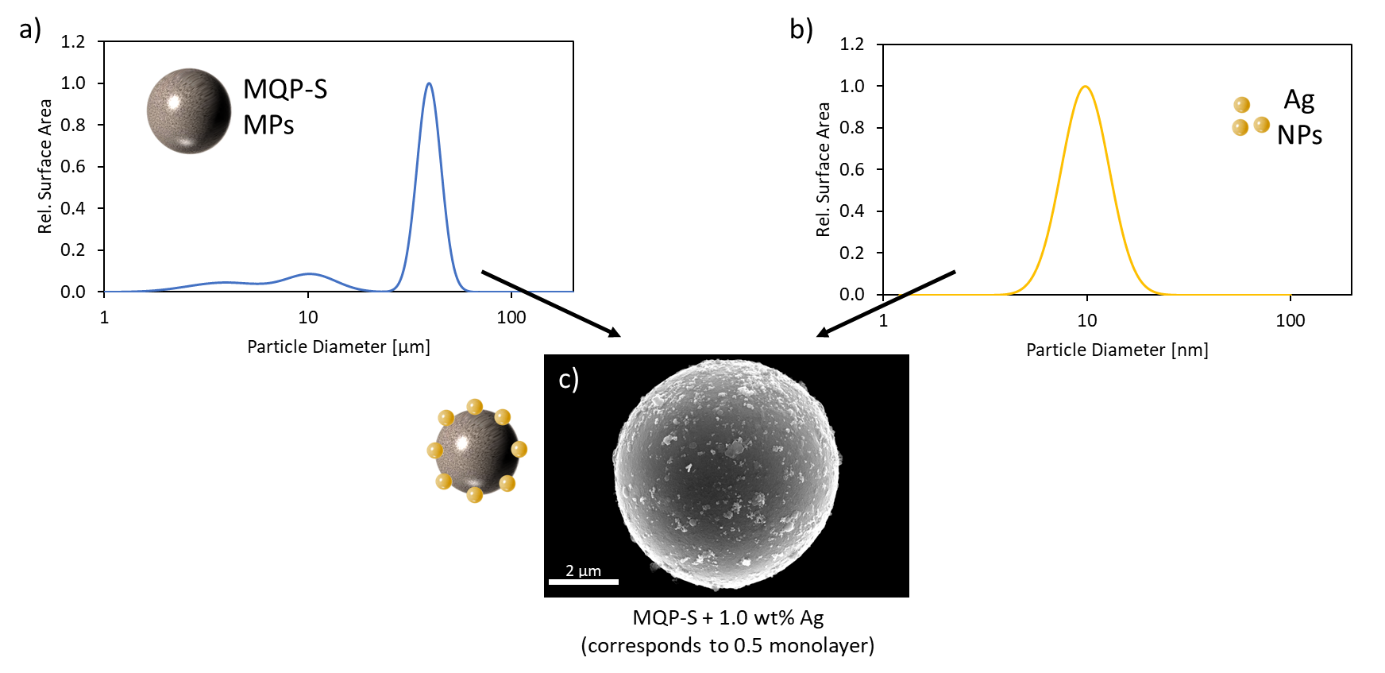


**Figure S1** Particle size distribution of a) MQP-S MPs and b) Ag NPs before additivation and c) SEM image after additivation showing a 0.5 monolayer with even distribution of Ag NP-islands. Sketches intend to show the surface modification in a simplified fashion (size relation and colors are not exact).

**2. Sample production via PBF-LB/M**

In total, 28 sample parts with a targeted diameter and height of 5 mm were produced by subjecting the unmodified MQP-S feedstock to PBF-LB/M while adjusting the laser power and scan speed, as shown in Figure S2. 15 samples have been used for further evaluation as reported in the main section of this study, while the additional 13 have been produced during the initial testing to determine the optimal process window. Also, with the Ag-modified feedstock, a total of 15 samples have been produced, as shown in Figure S3.


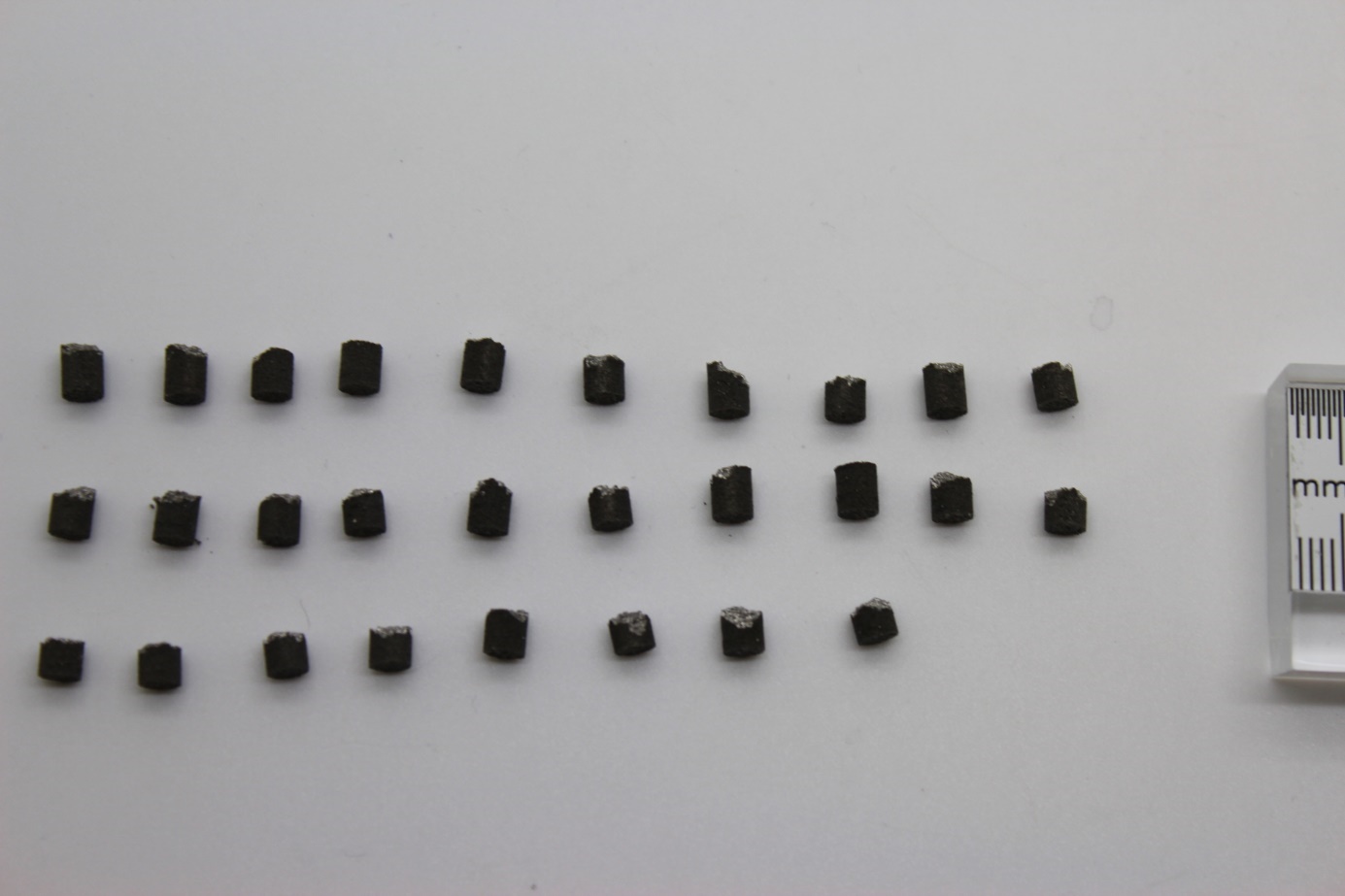


**Figure S2** Cylindrical samples (5 mm height, 5 mm diameter) produced of unmodified MQP-S feedstock via PBF-LB/M in the as-built condition.


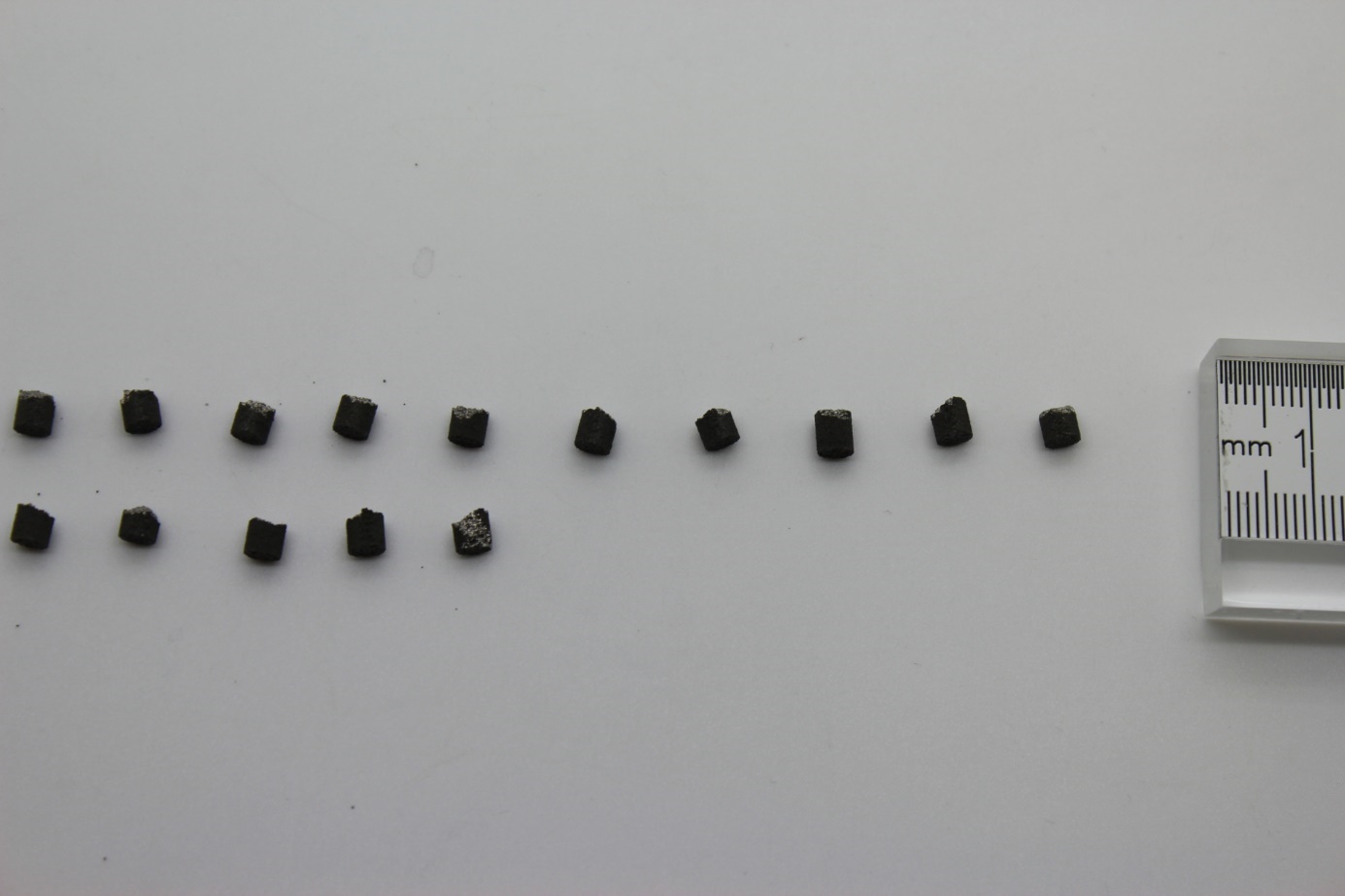


**Figure S3** Cylindric samples (5 mm height, 5 mm diameter) produced of MQP-S feedstock surface modified with 1 wt.% Ag NPs via PBF-LB/M in the as-built condition.

**3. Rich finite element simulation of the PBF-LB/M process to recapture melting**

**3.1 Non-isothermal phase field model for PBF-LB/M processes**

This model employs conserved and non-conserved phase-field order parameters (OPs) to represent the powder bed with multiple particles. The conserved OP $\rho$ indicates the substance (unmelted and melted region), whereby $\rho=1$ and $\rho=0$ represent the substance and atmosphere/pores, respectively. The non-conserved OPs ${\{}_{i}\}$ , $i=1, 2, \ldots$are used to distinguish particles with different crystallographic orientations. In each grain within the substance region, only one of ${}_{i}$ takes unity, while others have zero value ^[2,3]^. Thus, the numerical constraint $\sum_{i} {}_{i}+\left( 1- \rho\right)=1$ allows ${\{}_{i}\}$ to only be valued when $\rho=1$ i.e grains only exist within the substance region. A free energy functional is derived as

|  | $\left( T, \rho, {\{}_{i}\} \right)= \int_{\Omega} \left[ f\left( T, \rho, {\{}_{i}\} \right)+ \frac{1}{2}T\kappa_{\rho}{\vert\nabla\rho\vert}^{2}+ \frac{1}{2}T\kappa\sum_{i} {\vert\nabla{}_{i}\vert}^{2} \right]d\Omega,$ | (1) |
| --- | --- | --- |

where $T$ is the temperature, $\kappa_{\rho}$ and $\kappa$ are the gradient energy constants. The local free energy density $f\left( T, \rho, {}_{i} \right)$ is formulated in the form of a Landau-type polynomial as

|  | $\left( T, \rho, {\{}_{i}\} \right)= \Phi_{\mathrm{ht}}(\rho, {\{}_{i}\})f_{\mathrm{ht}}(T)+ \underline{C}(T)[\rho^{2}\left( 1-\rho\right)^{2}] + \underline{D}(T)\left[ \rho^{2}+6\left( 1-\rho\right)\sum_{i} \eta_{i}^{2}-4\left( 2-\rho\right)\sum_{i} \eta_{i}^{3}+3\left( \sum_{i} \eta_{i}^{2} \right)^{2} \right]$ | (2) |
| --- | --- | --- |

The local free energy reflects multiple minima, including $(\rho=0, {\{}_{1}= 0, {}_{2}= 0, {}_{N}= 0\})$ for atmosphere/pores region and $((\rho=1, {\{}_{1}= 1, {}_{2}= 0, {}_{N}= 0\}), (\rho=1, {\{}_{1}= 0, {}_{2}= 1, {}_{N}= 0\})$, … , $(\rho=1, {\{}_{1}= 0, {}_{2}= 0, {}_{N}= 1\})$ for grains with different orientations. $\underline{C}(T)$ and $\underline{D}(T)$ are temperature-dependent model parameters related to the barrier heights between these minima. Furthermore, model parameters $\underline{C}$ and $\underline{D}$ as well as $\kappa_{\rho}$ and $\kappa$ are directly related to temperature-dependent surface and grain boundary (GB) energies $\gamma_{\mathrm{sf}} (T)$ and $\gamma_{\mathrm{gb}} (T)$. $f_{\mathrm{ht}}(T)$ is the heat term that tilts the "multi-well" due to local heat variation, demonstrating the variable thermodynamic stability due to the change of local thermal conditions. $\Phi_{\mathrm{ht}}(\rho, {\{}_{i}\})$ maps $f_{\mathrm{ht}}(T)$to corresponding regions. Taking $T_{m}$ as reference temperature, $f_{\mathrm{ht}}$ has been derived as

|  | $f_{\mathrm{ht}} = c_{r}\left[ \left( T- T_{m} \right)- T\ln\frac{T}{T_{m}} \right]+ f_{\mathrm{ref}}^{T_{m}}- \Phi_{\mathrm{ml}}\frac{T - T_{m}}{T_{m}}L_{\mathrm{ht}}$. | (3) |
| --- | --- | --- |

where $f_{r\mathrm{ef}}^{T_{m}}$ is a referencing landscape of $f_{\mathrm{ht}}$ at $T_{m}$. $c_{r}$ is the relative specific heat that is directly related to the specific heat of the grains $c_{\mathrm{ss}}^{p}$ and atmosphere/pores $c_{\mathrm{at}}^{p}$. $L_{\mathrm{ht}}$ is the latent heat mapped by the interpolation function $\Phi_{\mathrm{ml}}$ ^[3,4]^.

The kinetics of the conserved and non-conserved OPs as well as that of $T$ can be derived based on the principles of non-equilibrium thermodynamics as presented by ^[2]^. Thereby the kinetics of the conserved OP $\rho$ can be formulated by the Cahn-Hilliard equation

|  | $\dot{\rho} =\nabla\cdot\left[ M\nabla\left( \frac{\partial f}{\partial\rho}-T\kappa_{\rho}\nabla^{2}\rho\right) \right]$. | (4) |
| --- | --- | --- |

$M$ is the mobility formulated to take into account mass transfer via multiple routes such as substance (ss), atmosphere (at), surface (sf), and grain boundary (gb), i.e.,

|  | $M = M_{\mathrm{ss}}^{\mathrm{eff}}\Phi_{\mathrm{ss}}\mathbf{I +}M_{\mathrm{at}}^{\mathrm{eff}}\Phi_{\mathrm{at}}\mathbf{I +}M_{\mathrm{sf}}^{\mathrm{eff}}\Phi_{\mathrm{sf}}\mathbf{T}_{\mathrm{sf}} + M_{\mathrm{gb}}^{\mathrm{eff}}\Phi_{\mathrm{gb}}\mathbf{T}_{\mathrm{gb}}+M_{\mathrm{ml}}^{\mathrm{eff}}\Phi_{\mathrm{ml}}\mathbf{T}_{\mathrm{sf}}$, | (5) |
| --- | --- | --- |

where $\Phi_{\mathrm{ss}}$, $\Phi_{\mathrm{at}}$, $\Phi_{\mathrm{sf}}$ and $\Phi_{\mathrm{gb}}$ are interpolation functions which obtain unity only in the corresponding region. $\mathbf{I}$ is the identity tensor while $\mathbf{T}_{\mathrm{sf}}$ and $\mathbf{T}_{\mathrm{gb}}$ are projection tensors projecting the mobilities onto the surface and grain boundaries respectively. Also, the mobilities $M_{\mathrm{ss}}^{\mathrm{eff}}$, $M_{\mathrm{at}}^{\mathrm{eff}}$, $M_{\mathrm{sf}}^{\mathrm{eff}}$ and $M_{\mathrm{gb}}^{\mathrm{eff}}$ can be directly obtained from the effective diffusivities $D_{\mathrm{ss}}^{\mathrm{eff}}$, $D_{\mathrm{at}}^{\mathrm{eff}}$, $D_{\mathrm{sf}}^{\mathrm{eff}}$ and $D_{\mathrm{gb}}^{\mathrm{eff}}$, respectively. $M_{\mathrm{ml}}^{\mathrm{eff}}$ effectively characterized the localized melt flow driven by the surface curvature, as elaborated in ^[55]^. In this work, $M_{\mathrm{ml}}^{\mathrm{eff}}$ is estimated to be $100M_{\mathrm{sf}}^{\mathrm{eff}}(T_{m})$.

On the other hand, the kinetics of ${\{}_{i}\}$ is governed by the Allen-Cahn equation with a corresponding mobility $L$ i.e.,

|  | $\dot{{}_{i}} =-L\left( \frac{\partial f}{\partial{}_{i}}-T\kappa\nabla^{2} {}_{i} \right)$ | (6) |
| --- | --- | --- |

It is important to note that the kinetics of ${\{}_{i}\}$ is reduced from Ref. ^[2]^, whereby temperature-gradient-driven GB migration is exempted. Also, mobility $L$ can be explicitly calculated using the GB mobility $G_{gb}^{eff}$, GB energy $\gamma_{\mathrm{gb}}$and the gradient energy parameter $\kappa$.

Finally, the heat transfer equation is formulated as

|  | $c_{r}\left[ \dot{T}-\mathbf{v}\cdot\nabla T \right]= \nabla\cdot\left( \mathbf{k}\cdot\nabla T \right)+q(\mathbf{r})$ | (7) |
| --- | --- | --- |

where it shows the coupling of $\dot{T}$ with the microstructure evolution. The thermal conductivity $\mathbf{k}$ adopts a similar formulation and spatial distribution as $\mathbf{M}$ i.e

|  | $\mathbf{M} = k_{\mathrm{ss}}^{\mathrm{eff}}\Phi_{\mathrm{ss}}\mathbf{I +}k_{\mathrm{at}}^{\mathrm{eff}}\Phi_{\mathrm{at}}\mathbf{I +}k_{\mathrm{sf}}^{\mathrm{eff}}\Phi_{\mathrm{sf}}\mathbf{T}_{\mathrm{sf}} + k_{\mathrm{gb}}^{\mathrm{eff}}\Phi_{\mathrm{gb}}\mathbf{T}_{\mathrm{gb}}$. | (8) |
| --- | --- | --- |

The heat generated by the scanning laser is modeled as a source term that follows the Gaussian surface distribution moving with a velocity $\mathbf{v}$**.** Also, the heat source term $q(\mathbf{r})$ indicates the volumetric energy deposition due to the radiative energy flux of the laser and is formulated as

|  | $q\left( \mathbf{r} \right)=Pp_{xy}[\mathbf{r}_{O}\left( \mathbf{v,t} \right)]\frac{da}{dz}$ | (9) |
| --- | --- | --- |

where $P$ is laser beam power and $\mathbf{v}$ is the laser scan velocity with its magnitude $v=|\mathbf{v|}$ taken as the scan speed. $p_{xy}$ is the Gaussian surface distribution with a moving center $\mathbf{r}_{O}\left( \mathbf{v,t} \right)$.

**3.2 Simulation setup and material properties**

The applied model is implemented numerically using the finite element method (FEM) within the "NIsoS" program developed by Yang et al. for non-isothermal phase-field models and simulations ^[2–4]^ based on the MOOSE framework ^[5,6]^. The meshing of the domain geometry is done using 8-node hexahedral Lagrangian elements. Within the framework, the transient solver with preconditioned Jacobian-Free Newton-Krylov method and backward Euler algorithm was used to solve the model's equations. Also, to reduce computational costs, adaptive meshing, and time-stepping schemes were employed. The Cahn-Hilliard equation is solved in a split way and the constraint of the OPs is ensured using a penalty function.

Material properties for Nd-Fe-B employed for the simulation are shown in Table S1. The thermal properties of the argon atmosphere are taken from Refs. ^[7,8]^. All parameters are normalized with the reference length and time scale of $\bar{l}=1 \mu m$, $\bar{t}=10 \mu s$, respectively. For the simulations, a $500\times250\times500 \mu m$ 3D domain is considered with no mass transfer allowed on all the boundaries, which is achieved by setting Neumann BC on $\rho$ as zero. The temperature at the bottom of the substrate mesh ($z_{\min}$) is fixed at room temperature $T_{0}=298 K$ via Dirichlet BC on $T$. Heat transfer is allowed only via the pore/atmosphere, achieved by the combined BC of convection and radiation, and masked by the interpolation function $\Phi_{\mathrm{at}}$. The powder bed with an average thickness of $\bar{h}=44 \mu m$ is placed on a substrate with the same composition and thickness of $250$ $\mu m$. The size distribution of the Nd-Fe-B powders is also reconstructed in log-normal with the arithmetic mean $\mu_{d}=32$ $\mu m$ and standard deviation $\sigma_{d}=8.67 \mu m$, fitted from the experimental measurements (see materials and methods section in the main manuscript).

**Table S1** Material properties of Nd-Fe-B employed in the simulations

| Properties | Expressions (T in K) | Units | References |
| --- | --- | --- | --- |
| $T_{m}$ | $1428$ | K | ^[9]^ |
| $\rho_{\mathrm{ss}}$ | $7410$ | kg m^-3^ | This study via pycnometry. |
| $k_{\mathrm{ss}}^{\mathrm{eff}}$ | $5.777 + 0.011T$ * | J s^-1^ m^-1^ K^-1^ | ^[10]^ |
| $c_{\mathrm{ss}}^{p}$ | $344.0218 + 0.3617T$ * | J kg^-1^ K^-1^ | ^[10]^ |
| $D_{\mathrm{ss}}^{\mathrm{eff}}$ | $2.17 \times{10}^{-5}exp(-2.717 \times{10}^{5} / RT$) | m² s^-1^ | ^[11]^ |
| $D_{\mathrm{gb}}^{\mathrm{eff}}$ | $2.40 \times{10}^{-3}exp(-1.770 \times{10}^{5} / RT$) | m² s^-1^ | ^[11]^ |
| $D_{\mathrm{sf}}^{\mathrm{eff}}$ | $0.4exp(-2.200 \times{10}^{5} / RT$) | m² s^-1^ | ^[12]^ |
| $G_{\mathrm{gb}}^{\mathrm{eff}}$ | $3.26 \times{10}^{-3}exp(-1.690 \times{10}^{5} / RT$) | m^4^ J^-1^ s^-1^ | ^[8]^ |
| $\gamma_{\mathrm{sf}}$ | $10.315-5.00 \times{10}^{-3} T$ | J m^-2^ | ^[13]^ |
| $\gamma_{\mathrm{gb}}$ | $13.018-7.50 \times{10}^{-3} T$ | J m^-2^ | ^[13]^ |
| $L_{\mathrm{ht}}$ | $2.4 \times{10}^{9}$ | J m^-3^ | ^[14]^ |

* Temperature-dependent data from Ref. ^[10]^ and scaled based on the value at $T=293K$

**Table S2** Volumetric energy densities (VED) of simulated process parameters that are shown in Figure 1 of the main section.

| Laser power [J s^-1^] | Scan speed [mm s^-1^] | Hatch distance [mm] | Layer thickness [mm] | VED [J mm^-^³] |
| --- | --- | --- | --- | --- |
| 50 | 1500 | 0.015 | 0.03 | 74.1 |
| 70 | 3000 | 0.015 | 0.03 | 51.9 |
| 74 | 2300 | 0.015 | 0.03 | 71.5 |
| 150 | 1700 | 0.015 | 0.03 | 196.1 |

**4. Functional properties of the as-built parts**


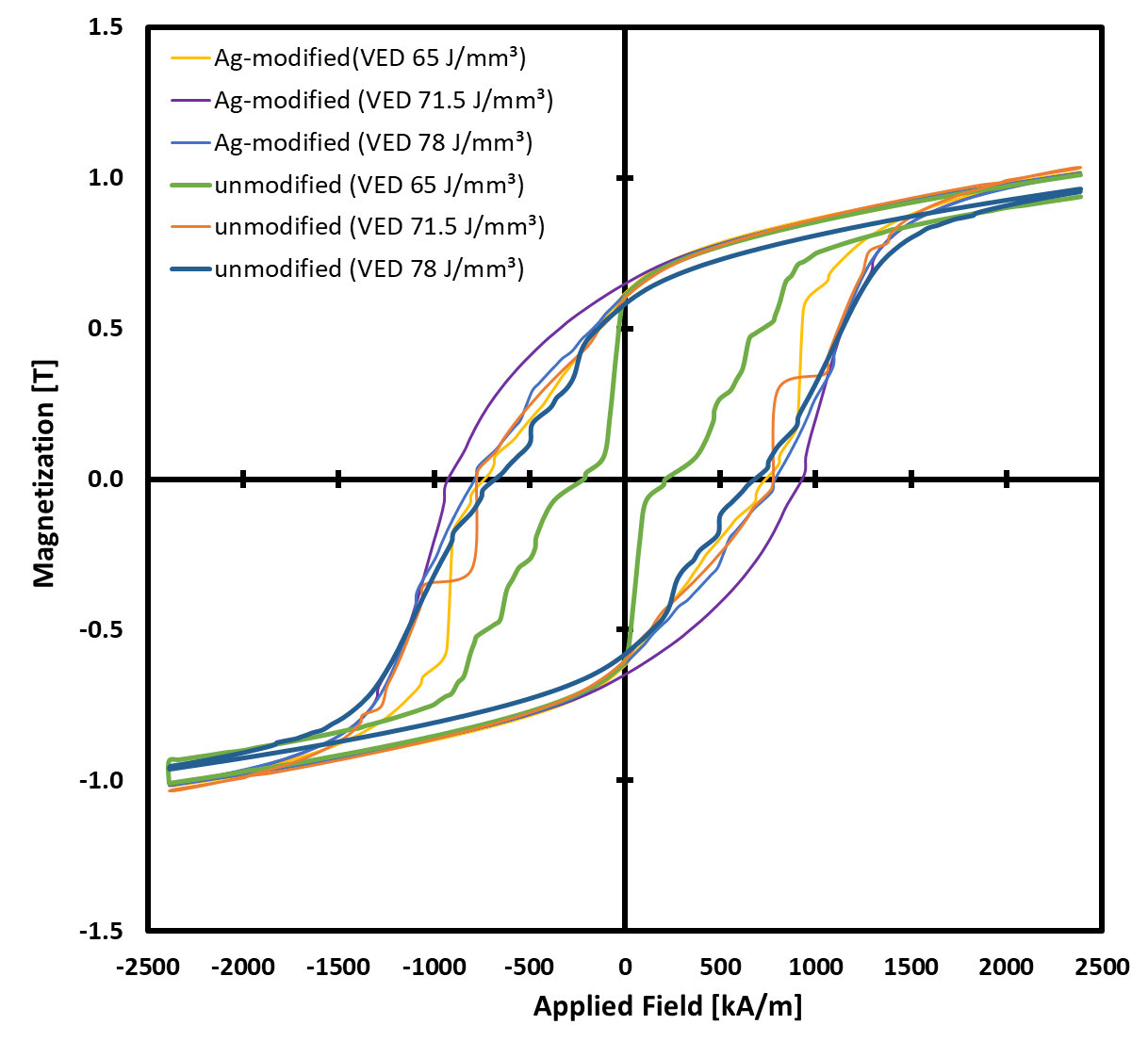


**Figure S4** Magnetic hysteresis loops of unmodified and Ag-modified MQP-S for different applied VEDs during the production of the magnet samples via PBF-LB/M.

**5. A literature review of achieved functional properties with PBF-LB/M of Nd-Fe-B-based feedstock**

Note that significant alterations of MQP-S or different compositions of Nd-Fe-B feedstock have not been included in the comparison with the results achieved in this study as they lack comparability but are to some extend discussed in the following paragraphs to give a thorough overview of the current state of research and indicate the differences between other studies and this study.

Since 2016, PBF-LB/M processing of the commercially available MQP-S powder has been studied by various groups ^[15–19]^. This is due to the spherical morphology of the powder enabling good powder flow behavior, which is very important for the PBF-LB/M process ^[20]^. Among them, Bittner et al. ^[21–23]^ optimized the process parameters to reduce porosity and stress cracks and achieved up to 921 kA m^-1^ coercivity, 0.63 T remanence, and 63 kJ m^-^³ energy product with a VED of 80 J mm^-^³. Huber et al. ^[19]^ used grain boundary (GB-) infiltration with low melting point eutectic alloys to further enhance the coercivity of PBF-LB/M processed MQP-S to 1211 kA m^-1^ by infiltration with Nd_50_Tb_20_Cu_30_ using very low VED of 0.21 J mm^-^³ but at the cost of increasing the RE-content and reducing the volume fraction of the 2-14-1 phase. The microstructure received by sintering (due to the very low applied VED) combined with the GB-infiltration led to a decreasing of the remanence to 0.39 T. To our knowledge, this is the only study using such low VED that leads to the sintering of the MQP-S powder particles instead of melting. Furthermore, Yao et al. ^[24]^ demonstrated that the magnetic properties (VIM: H_c_ of 108 kA m^-1^, B_r_ of 0.57 T; LDED: H_c_ of 95 kA m^-1^, B_r_ of 0.57 T; PBF-LB/M: H_c_ of 656 kA m^-1^, B_r_ of 0.79 T), microhardness (VIM: 444 HV; LDED: 741 HV; PBF-LB/M: 893 HV), and microstructure formation of Nd-Fe-B-based permanent magnets produced via PBF-LB/M are superior to those produced through vacuum induction melting (VIM) and laser directed energy deposition (LDED) methods, which they mainly referred to the significantly different cooling rates.

Another approach of several groups was to alter the commercial MQP-S powder composition by alloying different amounts of highly magnetic elements or alloys, e.g., the addition of 20% of (NdPr)_3_Cu_0.25_Co_0.75_ to MQP-B (an alternative powder to MQP-S, based on a NdFeCoB composition typically used for manufacturing of bonded magnets) and reached a coercivity of 1250 kA m^-1^ ^[25]^. Or by using (over-)stoichiometric powder compositions with 18 at.% RE ^[26]^, RE-rich Nd_21_Fe_73.5_Cu_2_B_3.5_ and Pr_21_Fe_73.5_Cu_2_B_3.5_ ^[27]^, or Pr_20.5_Fe_73.8_Cu_2.0_B_3.7_ ^[28]^, which are known from conventional sintering processes to improve the magnetic properties. The magnetic properties can be improved by applying further post-processing after PBF-LB/M with one or multiple heat treatments. Goll et al. ^[26]^ demonstrated with Nd_16.5_Pr_1.5_Zr_2.6_Ti_2.5_Co_2.2_Fe_65.9_B_8.8_ the possibility to increase the magnetic properties from 420 kA m^-1^, 0.57 T, 48 kJ m^-^³ to
925 kA m^-1^, 0.58 T, 62.3 kJ m^-^³ by applying a two-step heat treatment of 600°C for 10 min followed by 500°C for 60 min.

Tosoni et al. ^[29]^ recently developed a Cu-rich Nd-Fe-B powder (Nd_12.1_Pr_0.8_Dy_1.5_Fe_bal._B_6.3_Co_1.24_Al_1_Cu_1_Ga_0.2_), with a composition otherwise close to Dy-doped sintered magnets for PBF-LB/M processing, which lead to B_r_ = 0.58 T, H_c_ = 1210 kA m^-1^ and BH_max_ = 48 kJ m^-^³ in as-built condition. After a two-step post-process annealing (600°C, 10 min + 470°C, 60 min) they achieved magnetic properties of B_r_ = 0.62 T, H_c_ = 1790 kA m^-1^ and BH_max_ = 65 kJ m^-^³. So far, this represents the highest ever reported coercivity for additively manufactured Nd-Fe-B, but at the cost of increasing the RE content to 14.6 at.% (compared to 8.2 at.% of MQP-S).

Post-process annealing enhances magnetic properties (especially coercivity) by improving the formation and wetting of the RE-rich grain boundary phase required for magnetic decoupling of the Nd_2_Fe_14_B grains, which seems not to happen sufficiently during the PBF-LB/M process, possibly due to the high cooling rates of 10³ - 10^5^ K s^-1^ ^[30]^ leading to rapid solidification. Note that significant changes in microstructure have been observed after very long annealing treatments (5 h at 1000°C + 3 h at 500°C) by the formation of small grains of 2:14:1 phases, but also a formation of intermetallic non-magnetic phases like e.g. (Nd, Pr)_6_Fe_13_Cu phase (denoted as 6:13:1)^[27]^. After annealing, small grains of binary Nd_2_Fe_17_ and Pr_2_Fe_17_ phases (denoted as 2:17) were also observed.^[27]^

The aforementioned publications and our best-performing results are summarized and compared in Table S3. As stated above, some of the publications performed heat treatments, and not all used commercially available MQP-S powder feedstock. To enable better comparability, we investigated our samples directly after the PBF-LB/M process without further heat treatment, and only the achieved results in as-built condition after PBF-LB/M with MQP-S powder are included in the further comparison here.

**Table S3** Overview of achieved magnetic properties of samples produced via PBF-LB/M and applied VED with MQP-S feedstock, sorted from lowest to highest applied VED. No results achieved by heat treatments after the PBF-LB/M process are included. The numbering of the literature references is aligned to the reference list given in the main manuscript (not the reference list of the Supporting Information).

| No. | References  (as in the manuscript) | Coercivity [kA/m] | Remanence  [T] | Laser Power [W] | Scan Speed [mm/s] | VED [J/mm³] |
| --- | --- | --- | --- | --- | --- | --- |
| 1 | [14] | 516 | 0.56 | 60 | 160 | 18.75 |
| 2 | [24] | 656 | 0.79 | 120 | 1200 | 33.33 |
| 3 | [11] | 603 | 0.65 | 100 | 903 | 36.91 |
| 4 | [23] | 603 | 0.62 | 100 | 903 | 36.91 |
| 5 | [17] | 885 | 0.63 | 125 | 1000 | 55.56 |
| 6 | [16] | 420 | 0.57 | 200 | 2000 | 66.67 |
| 7.1 | this study (unmodified) | 769 | 0.51 | 74 | 2300 | 71.50 |
| 7.2 | this study (Ag-modified) | 935 | 0.545 | 74 | 2300 | 71.50 |
| 8 | [12] | 695 | 0.59 | 68 | 360 | 94.44 |
| 9 | [13] | 871 | 0.56 | 55 | 1400 | 98.21 |
| 10 | MQP-S datasheet*^[34]^ | 730* | 0.75* | NA | NA | NA |

*not achieved via PBF-LB/M, but measured in the raw powder according to the manufacturer.


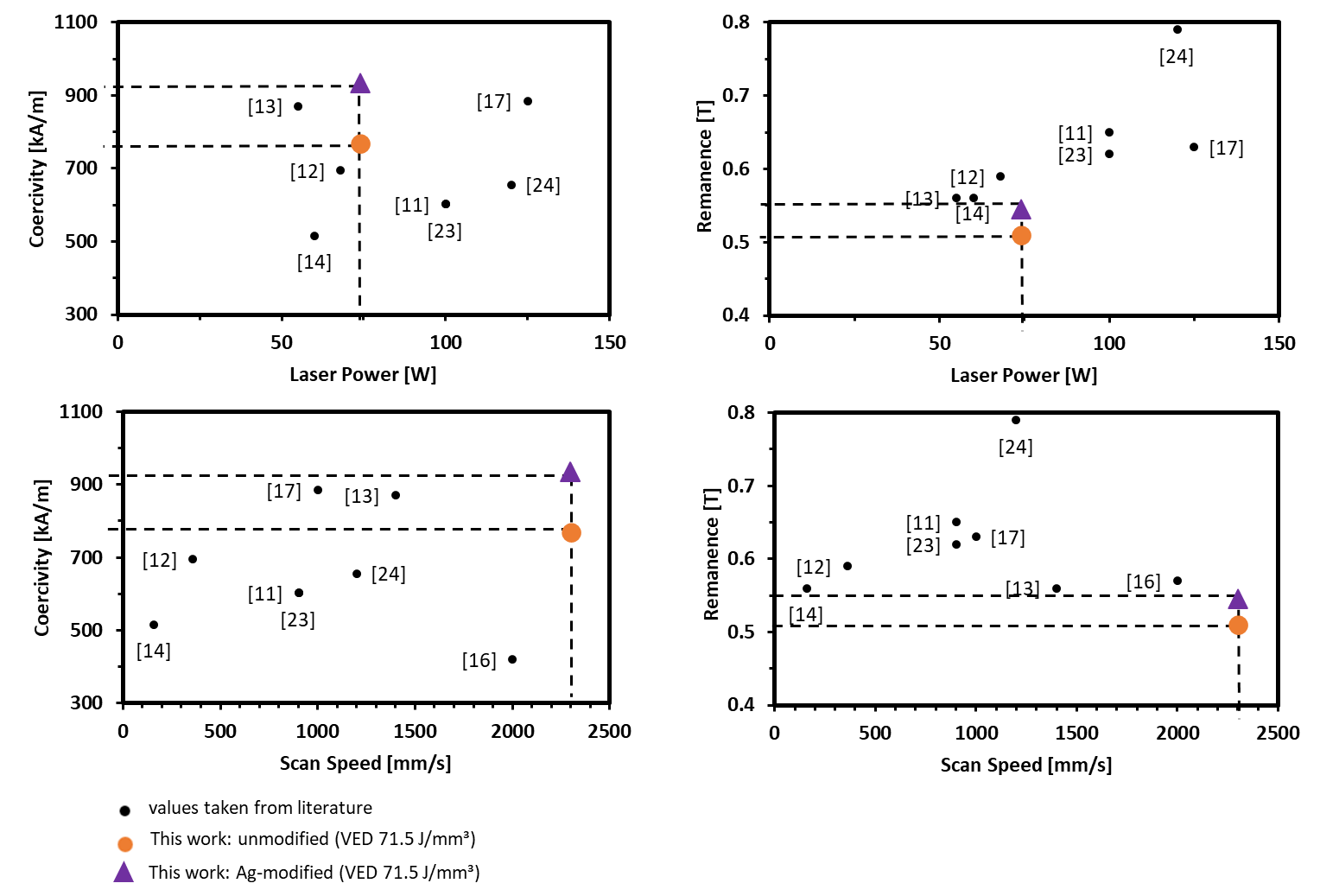


**Figure S5** Laser power, scan speed, and achieved magnetic properties of references (labeling as declared in Table S3) in comparison to our results, where the lower orange circle indicates the achieved properties of unmodified samples and the upper purple triangle indicates the achieved results of Ag-modified samples. No results achieved by heat treatments after the PBF-LB/M process are included. The numbering of the literature references is aligned to the reference list given in the main manuscript (not the reference list of the Supporting Information).

**6. Microstructural and compositional analysis**

Two different contrasts can be seen in the SEM images corresponding to the primary Nd_2_Fe_14_B phase appearing grey and the Nd-rich precipitates with a bright contrast. The Nd-rich precipitates appear in both the unmodified and Ag-modified samples


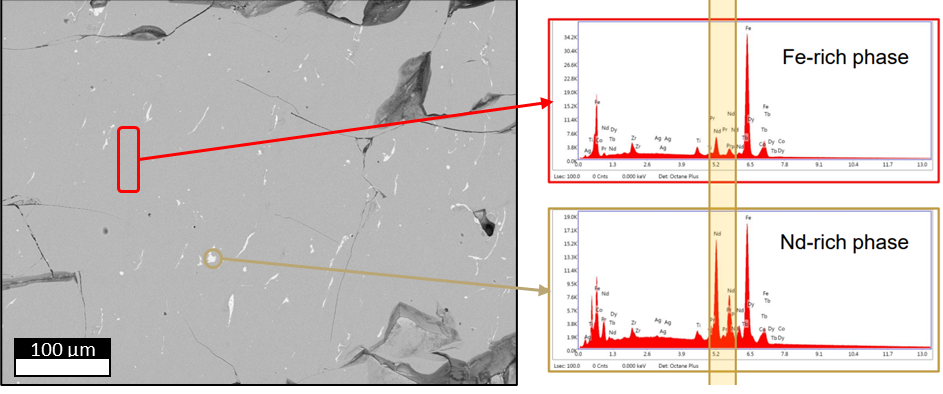


**Figure S6** EDS analysis of an unmodified sample with the chemical spectra of the Fe-rich (Nd-Fe-B) and the Nd-rich precipitates, respectively, for two exemplary areas (clearly differentiable by the Nd content highlighted in the yellow bar).


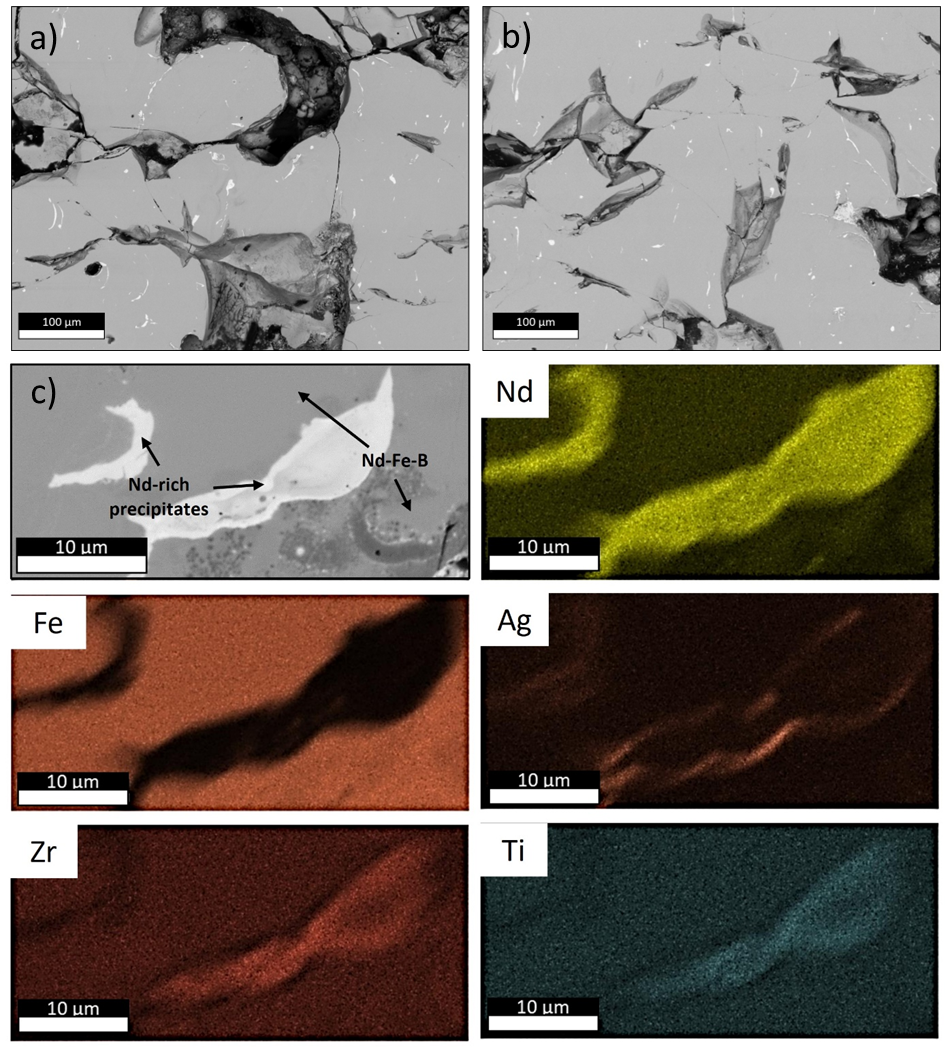


**Figure S7** Microstructural analysis of Ag-modified samples. a), b) SEM images of cross-sections (X-Y plane), c) Higher magnification SEM image and EDS elemental mappings highlighting the distribution of individual elements between the primary Nd-Fe-B (2:14:1) and the Nd-rich precipitates.


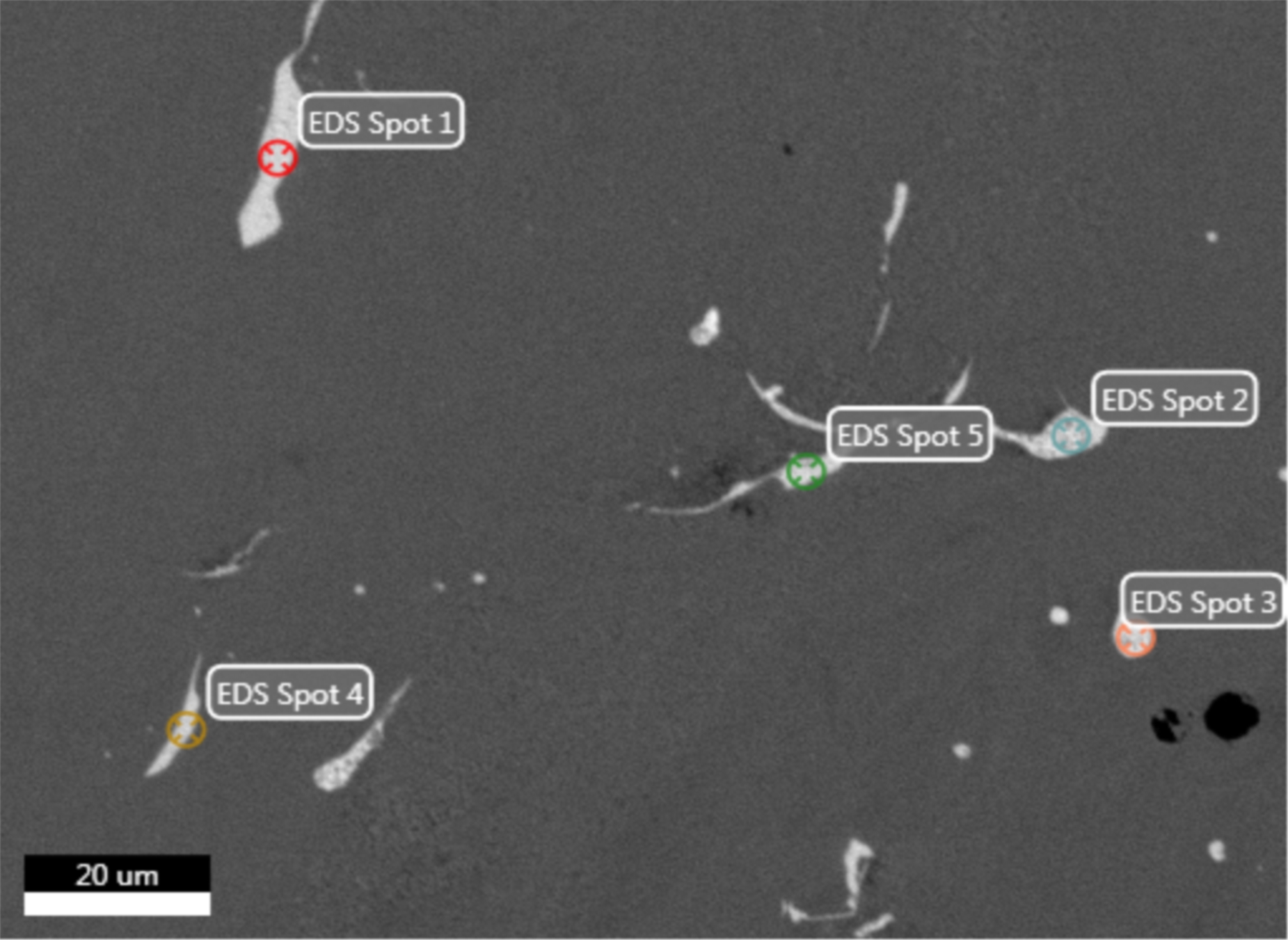


**Figure S8** SEM micrograph showing EDS analysis points selected for composition calculation of the Nd-rich phase in an unmodified sample.

**Table S4** Elemental composition of the EDS point analysis shown in Figure S8 of an unmodified sample

| Element | Nd  [at.%] | Fe  [at.%] | Co  [at.%] | Pr  [at.%] | Zr  [at.%] | Ti  [at.%] |
| --- | --- | --- | --- | --- | --- | --- |
| EDS Spot 1 | 7.04 ± 0.63 | 82.14 ± 3.34 | 3.08 ± 0.80 | 1.01 ± 0.30 | 3.05 ± 0.23 | 3.26 ± 0.35 |
| EDS Spot 2 | 7.46 ± 0.65 | 83.61 ± 3.37 | 2.97 ± 0.68 | - | 2.26 ± 0.17 | 3.54 ± 0.35 |
| EDS Spot 3 | 7.19 ± 0.62 | 82.64 ± 3.36 | 3.08 ± 0.81 | - | 2.91 ± 0.22 | 3.85 ± 0.35 |
| EDS Spot 4 | 7.62 ± 0.68 | 82.52 ± 3.32 | 3.04 ± 0.76 | - | 2.88 ± 0.23 | 3.84 ± 0.35 |
| EDS Spot 5 | 5.02 ± 0.48 | 86.98 ± 3.57 | 2.56 ± 0.80 | - | 2.18 ± 0.16 | 3.12 ± 0.32 |


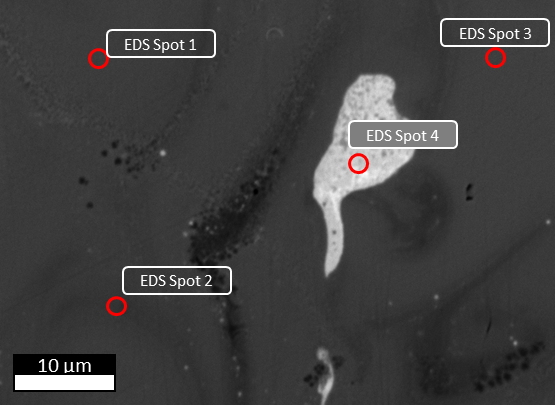


**Figure S9** SEM micrograph showing EDS analysis points selected for composition calculation of an Ag-modified sample.

**Table S5** Elemental composition of the EDS point analysis shown in Figure S9 of an Ag-modified sample

| Element | Ag [at.%] | Nd [at.%] | Fe [at.%] | Co [at.%] | Pr [at.%] | Zr [at.%] | Ti [at.%] |
| --- | --- | --- | --- | --- | --- | --- | --- |
| EDS Spot 1 | 0.14 ± 0.07 | 6.70 ± 0.40 | 83.21 ± 2.25 | 2.26 ± 0.41 | 0.81 ± 0.17 | 3.01 ± 0.15 | 3.21 ± 0.21 |
| EDS Spot 2 | 0.31 ± 0.11 | 5.57 ± 0.34 | 83.31 ± 2.40 | 2.79 ± 0.45 | 0.77 ± 0.16 | 3.00 ± 0.14 | 3.22 ± 0.20 |
| EDS Spot 3 | 0.29 ± 0.16 | 7.13 ± 0.40 | 81.84 ± 2.37 | 2.49 ± 0.44 | 0.95 ± 0.19 | 2.82 ± 0.14 | 3.23 ± 0.21 |
| EDS Spot 4 | 5.66 ± 0.39 | 68.43 ± 1.91 | 12.06 ± 0.93 | 0.38 ± 0.38 | 7.87 ± 0.72 | 3.40 ± 0.33 | 1.18 ± 0.30 |


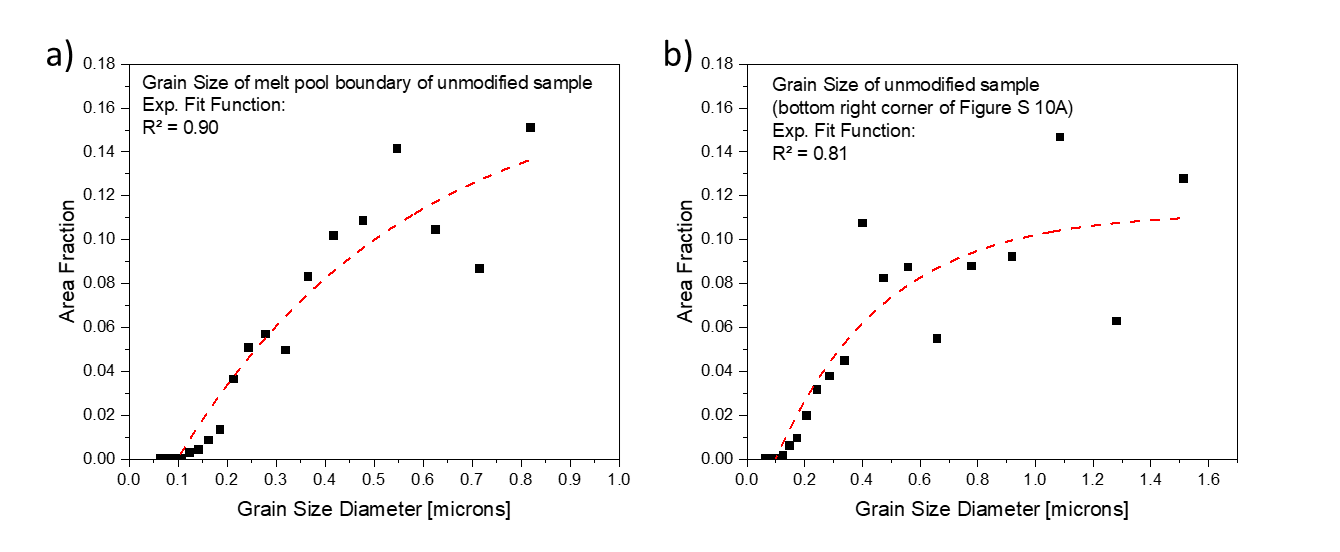


**Figure S10** Grain size analysis from EBSD of the unmodified sample shown in Figure S10a; a) in the melt pool boundary region and b) in the lower right corner.

**Figure S11** Grain size analysis from EBSD of the Ag-modified sample based on EBSD analysis shown in Figure S10b.


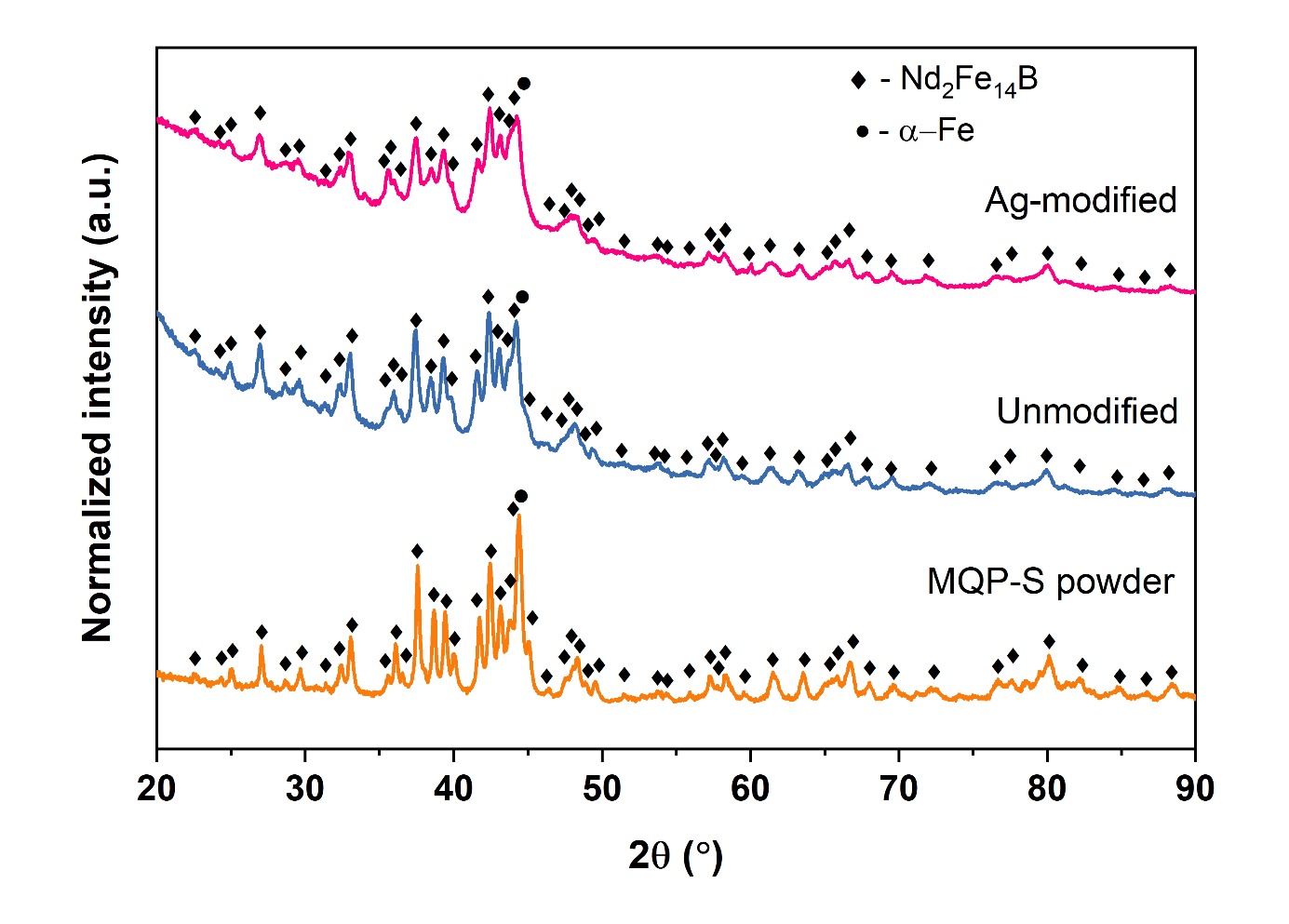


**Figure S12** X-ray diffractograms of the MQP-S feedstock powder, unmodified and Ag-modified as-built parts. The XRD patterns highlight the presence of the primary phase being tetragonal Nd_2_Fe_14_B (space group: P4_2_/mnm, lattice parameters: a = 0.876 nm and c = 1.211 nm, ICSD database code: 68609) along with a reflex from the α-Fe phase. The intensity of the α-Fe phase is drastically reduced in the as-built parts signifying the predominant formation of Nd_2_Fe_14_B under fast cooling conditions.


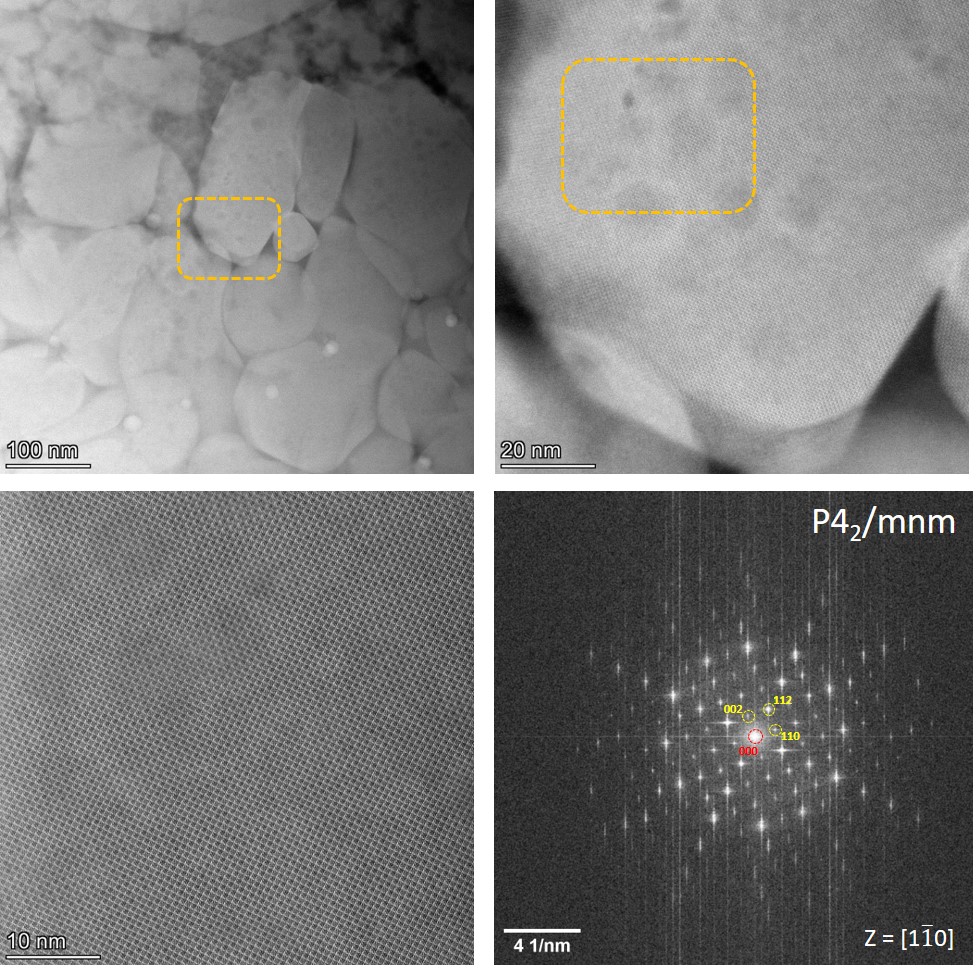


**Figure S13** STEM-HAADF micrographs and respective FFT analysis of the unmodified as-built sample revealing Nd-Fe-B (2:14:1) crystallites surrounded by an amorphous phase. Indexing of the FFT confirms the phase to be tetragonal Nd_2_Fe_14_B (space group P4_2_/mnm).


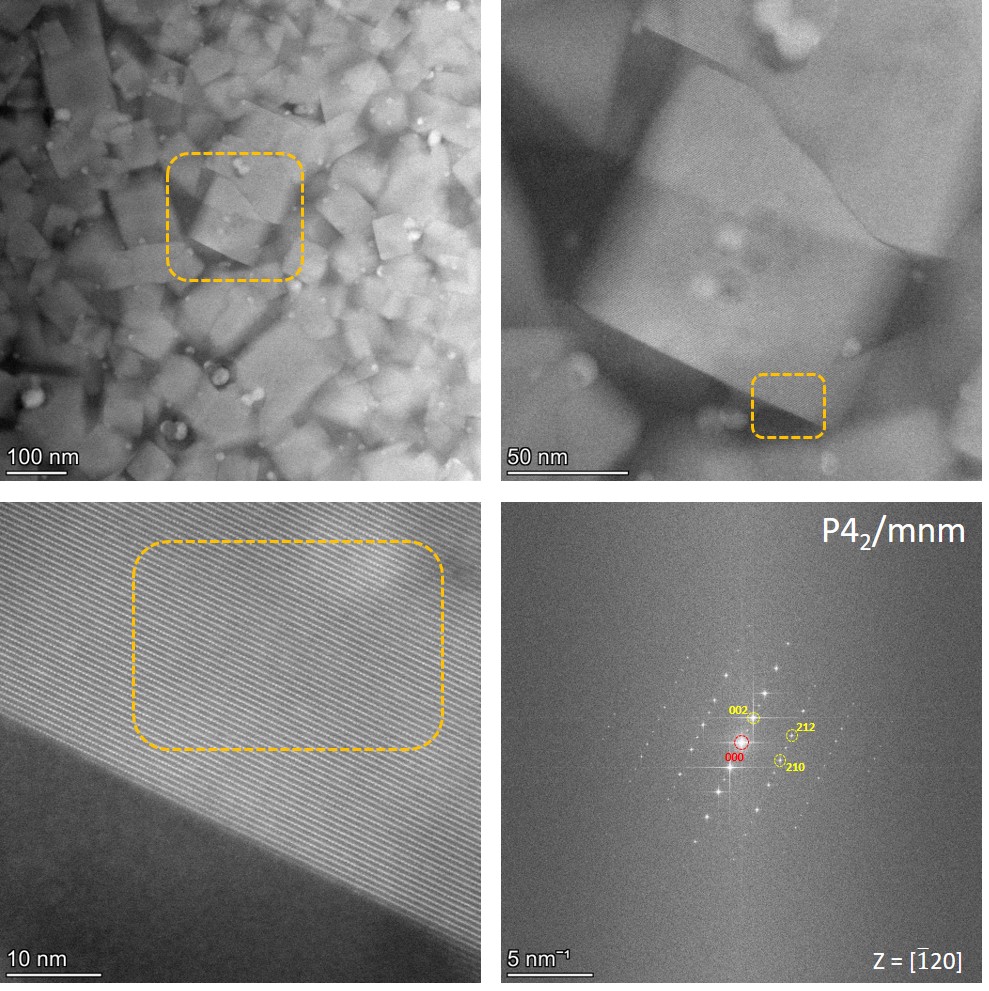


**Figure S14** STEM-HAADF micrographs and respective FFT analysis of the Ag-modified as-built sample revealing Nd-Fe-B (2:14:1) crystallites surrounded by an amorphous phase. Indexing of the FFT confirms the phase to be tetragonal Nd_2_Fe_14_B (space group P4_2_/mnm).


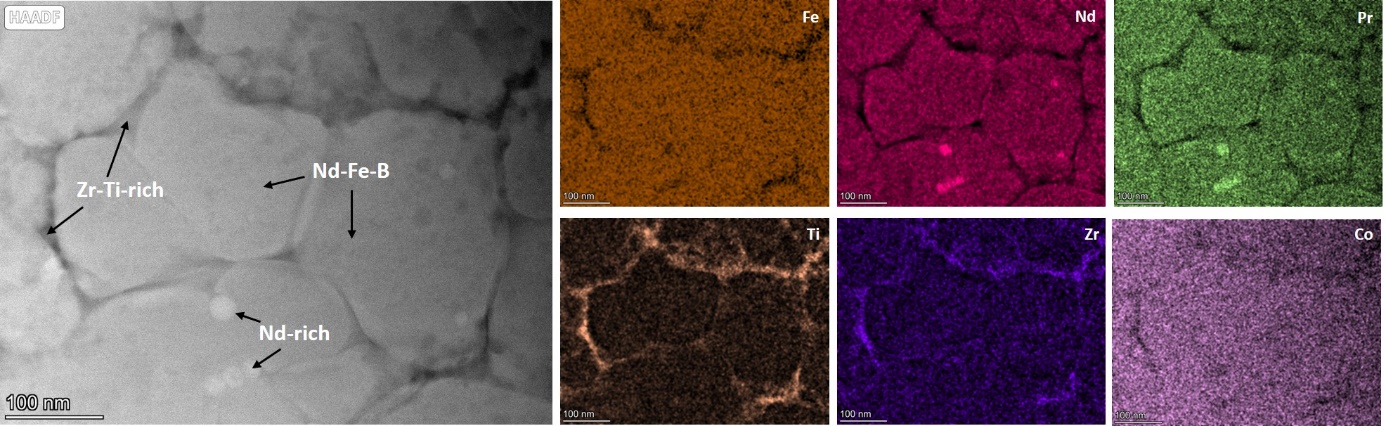


**Figure S15** STEM-EDS mapping of the unmodified sample.


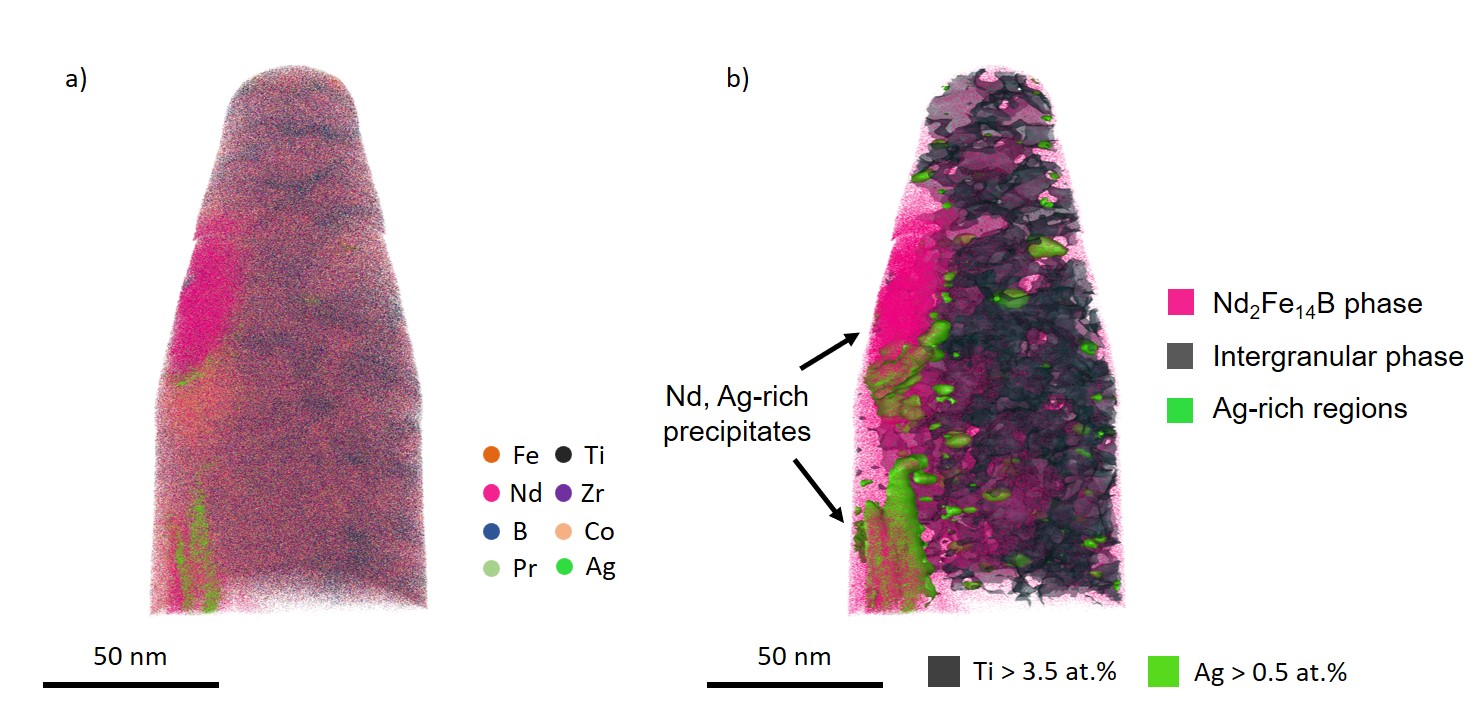


**Figure S16** APT results of the Ag-modified as-built sample. a) 3D-reconstructed APT data; b) 3D atom map showing the intergranular phase with the isosurface of Ti (grey) greater than 3.5 at.% and the Ag distribution with the isosurface of Ag (light green) greater than 0.5 at.%.

**Table S6** Average chemical composition of the different phases detected via APT in the Ag-modified sample.

| Element | Fe  [at.%] | Nd  [at.%] | B  [at.%] | Co  [at.%] | Zr  [at.%] | Ti  [at.%] | Pr  [at.%] | Ag  [at.%] |
| --- | --- | --- | --- | --- | --- | --- | --- | --- |
| Phase 1 (purple) Nd_2_Fe_14_B phase | 76.07 ± 1.45 | 7.38 ±  0.90 | 5.6 ± 0.79 | 2.67 ± 0.55 | 2.74 ± 0.56 | 1.79 ± 0.46 | 0.76 ± 0.32 | - |
| Phase 2 (grey) Intergranular phase | 64.48 ± 2.13 | 0.61 ± 0.35 | 12.9 ± 1.49 | 2.38 ± 0.68 | 3.57 ± 0.82 | 9.33 ± 1.30 | 0.21 ± 0.17 | 0.03 ± 0.03 |
| Phase 3 (green) Ag-rich region | 70.40 ± 1.53 | 6.61 ± 0.83 | 6.83 ± 0.84 | 2.46 ± 0.51 | 2.58 ± 0.53 | 3.14 ± 0.58 | 0.56 ± 0.25 | 2.8 ± 0.55 |

Trace amounts of Cu, C, Cr, Al, P, O, and Si were in the remaining.

**References of the Supporting Information**

[1] A. Fojtik, A. Henglein, *Berichte Bunsen-Ges.-Phys. Chem. Chem. Phys.* **1993**, *97*, 252.

[2] Y. Yang, T. D. Oyedeji, P. Kühn, B.-X. Xu, *Scr. Mater.* **2020**, *186*, 152.

[3] Y. Yang, O. Ragnvaldsen, Y. Bai, M. Yi, B.-X. Xu, *Npj Comput. Mater.* **2019**, *5*, 1.

[4] Y. Yang, T. D. Oyedeji, X. Zhou, K. Albe, B.-X. Xu, *Npj Comput. Mater.* **2023**, *9*, 1.

[5] C. J. Permann, D. R. Gaston, D. Andrš, R. W. Carlsen, F. Kong, A. D. Lindsay, J. M. Miller, J. W. Peterson, A. E. Slaughter, R. H. Stogner, R. C. Martineau, *SoftwareX* **2020**, *11*, 100430.

[6] M. R. Tonks, D. Gaston, P. C. Millett, D. Andrs, P. Talbot, *Comput. Mater. Sci.* **2012**, *51*, 20.

[7] Y. Yang, P. Kühn, M. Yi, H. Egger, B.-X. Xu, *JOM* **2020**, *72*, 1719.

[8] Y. Yang, O. Ragnvaldsen, Y. Bai, M. Yi, B.-X. Xu, *Npj Comput. Mater.* **2019**, *5*, 81.

[9] C. Rong, B. Shen, *Chin. Phys. B* **2018**, *27*, 117502.

[10] J. J. Valencia, P. N. Quested, in *Cast. Vol 15 ASM Handb. ASM Int.*, **2013**.

[11] J. M. Blakely, H. Mykura, *Acta Metall.* **1963**, *11*, 399.

[12] H. W. Mead, C. E. Birchenall, *JOM* **1956**, *8*, 1336.

[13] A. T. Price, H. A. Holl, A. P. Greenough, *Acta Metall.* **1964**, *12*, 49.

[14] F. R. Liu, Q. Zhang, W. P. Zhou, J. J. Zhao, J. M. Chen, *J. Mater. Process. Technol.* **2012**, *212*, 2058.

[15] J. Wu, N. T. Aboulkhair, M. Degano, I. Ashcroft, R. J. M. Hague, *Mater. Des.* **2021**, *209*, 109992.

[16] J. Jaćimović, F. Binda, L. G. Herrmann, F. Greuter, J. Genta, M. Calvo, T. Tomše, R. A. Simon, *Adv. Eng. Mater.* **2017**, *19*, 1700098.

[17] N. Urban, A. Meyer, V. Keller, J. Franke, *Appl. Mech. Mater.* **2018**, *882*, 135.

[18] M. Skalon, M. Görtler, B. Meier, S. Arneitz, N. Urban, S. Mitsche, C. Huber, J. Franke, C. Sommitsch, *Materials* **2020**, *13*, 139.

[19] C. Huber, H. Sepehri-Amin, M. Goertler, M. Groenefeld, I. Teliban, K. Hono, D. Suess, *Acta Mater.* **2019**, *172*, 66.

[20] U. Scipioni Bertoli, A. J. Wolfer, M. J. Matthews, J.-P. R. Delplanque, J. M. Schoenung, *Mater. Des.* **2017**, *113*, 331.

[21] F. Bittner, J. Thielsch, W.-G. Drossel, *Prog. Addit. Manuf.* **2020**, *5*, 3.

[22] F. Bittner, J. Thielsch, W.-G. Drossel, *Scr. Mater.* **2021**, *201*, 113921.

[23] F. Bittner, J. Thielsch, W.-G. Drossel, *IEEE Trans. Magn.* **2022**, *58*, 1.

[24] B. Yao, N. Kang, X. Li, D. Li, M. E. Mansori, J. Chen, H. Yang, H. Tan, X. Lin, *Int. J. Extreme Manuf.* **2023**, *6*, 015002.

[25] A. S. Volegov, S. V. Andreev, N. V. Selezneva, I. A. Ryzhikhin, N. V. Kudrevatykh, L. Mädler, I. V. Okulov, *Acta Mater.* **2020**, *188*, 733.

[26] D. Goll, F. Trauter, T. Bernthaler, J. Schanz, H. Riegel, G. Schneider, *Micromachines* **2021**, *12*, 538.

[27] L. Schäfer, K. Skokov, J. Liu, F. Maccari, T. Braun, S. Riegg, I. Radulov, J. Gassmann, H. Merschroth, J. Harbig, M. Weigold, O. Gutfleisch, *Adv. Funct. Mater.* **2021**, *31*, 2102148.

[28] D. Goll, F. Trauter, R. Loeffler, T. Gross, G. Schneider, *Micromachines* **2021**, *12*, 1056.

[29] O. Tosoni, E. Borges Mendonça, J. Reijonen, A. Antikainen, L. Schäfer, S. Riegg, O. Gutfleisch, *Addit. Manuf.* **2023**, *64*, 103426.

[30] P. A. Hooper, *Addit. Manuf.* **2018**, *22*, 548.
